# Dual Randomness From Spatial and Temporal Variations in Stochastic Zinc Gallate Nanoclusters for PUF and TRNG Co‐Driven Image Generation

**DOI:** 10.1002/advs.78054

**Published:** 2026-09-28

**Authors:** Seungme Kang, Wangmyung Choi, Jueun Lee, Yeong Kwon Kim, Byung Chul Jang, Wonjun Shin, Hocheon Yoo

**Affiliations:** ^1^ Department of Electronic Engineering Hanyang University Seoul Republic of Korea; ^2^ Department of Electrical and Computer Engineering Sungkyunkwan University (SKKU) Suwon Republic of Korea; ^3^ School of Electronic and Electrical Engineering Kyungpook National University Daegu Republic of Korea; ^4^ Department of Semiconductor Convergence Engineering Sungkyunkwan University (SKKU) Suwon Republic of Korea; ^5^ Department of Artificial Intelligence Semiconductor Engineering Hanyang University Seoul Republic of Korea; ^6^ Hanyang Institute for Quantum Science and Quantum Technology Hanyang University Seoul Republic of Korea

**Keywords:** device‐to‐device variation, generative adversarial network, physical unclonable function, time‐to‐noise variation, true random number generator, zinc gallate nanocluster

## Abstract

We propose a novel dual‐randomness framework that integrates physically unclonable functions (PUFs) and true random number generators (TRNGs) within a single nanomaterial platform. In contrast to conventional approaches that treat PUFs and TRNGs as separate entities, our platform integrates both entropy sources within a single stochastic nanomaterial system, enabling parallel and co‐localized operation. The mixed zinc gallate (ZGO) nanoclusters (NCs) present stochastic properties in which size‐dependent variations influence device resistance, capacitance, and noise characteristics. These variations lead to unique device signatures, while time‐dependent noise fluctuations serve as a TRNG source. The proof as a PUF is confirmed by the difference in position through the measurement of the work function, capacitance, and impedance. The device‐derived TRNG bitstreams from 20 devices exceeded the acceptance threshold of 0.01 for all 11 reported NIST criteria. By integrating the stochastic characteristics arising from the spatially dependent and time‐dependent properties from mixed ZGO NCs, the dilemma of a single device is solved. To demonstrate hardware‐derived latent‐vector modulation, experimentally measured current traces from the mixed‐ZGO devices were converted into a 512‐dimensional latent vector and supplied to a pretrained StyleGAN2‐AFHQv2 generator.

## Graphical abstract

A dual‐randomness hardware platform is demonstrated by integrating PUF and TRNG functionalities into mixed‐ZGO nanoclusters. By leveraging spatial and temporal stochasticity, the mixed‐ZGO NCs device enables both unique identification and random signal generation. The extracted entropy is applied to a StyleGAN2‐AFHQv2 framework, generating various and unpredictable animal images.

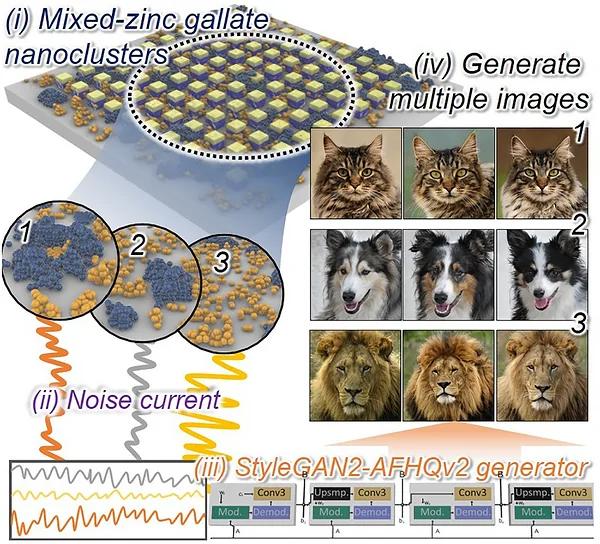

## 1 Introduction

Physical unclonable functions (PUFs) have emerged as a critical technology for hardware security, adopting inherent process variations to generate unique and unclonable identifiers [1, 2, 3]. Conventional PUF implementations predominantly rely on complementary metal‐oxide‐semiconductor (CMOS) silicon‐based architectures, such as static random access memory (SRAM) [4, 5], dynamic random access memory (DRAM) [6, 7], NAND‐flash memory [8, 9], or via‐based designs [10, 11], where random process variations in semiconductor fabrication define the device‐specific response. These methods exploit random process variations occurring during semiconductor fabrication to generate unique challenge‐response pairs (CRPs). While effective, these circuit‐based approaches are constrained by their reliance on predefined semiconductor structures and suffer from circuit complexity and accordingly, high‐cost fabrication process.

To address these limitations, recent advancements in material‐based PUFs have explored employing intrinsic morphological variations in materials to generate randomness at a fundamental level [12, 13, 14]. Rather than circuit‐based approaches, these methods utilize stochastic variations arising from material synthesis, as follows: blending multiple materials [15, 16], structural heterogeneity [17, 18, 19], defect‐induced randomness [20, 21, 22], electronic disorder at the interface employing co‐evaporation techniques [23], integrating particles [3, 24] or optical materials [25, 26, 27], and employing atomic‐scale disorder in two‐dimensional materials [28, 29], grain boundaries, and charge trap sites [30, 31, 32]. These techniques introduce low‐level physical disorder: material‐induced stochasticity that is inherently uncontrollable and unpredictable, thereby enabling PUF implementations that offer higher security and resilience against physical attacks. In addition to its effectiveness, material‐based randomness eliminates the need for complex circuit designs, reducing fabrication costs and expanding PUF applicability beyond conventional silicon‐based methodologies.

Compared to the above‐mentioned PUFs, which generate finite sets of CPR based on structural randomness, true random number generators (TRNGs) exploit temporal variations to produce an endless stream of unpredictable yet uncontrollable outputs [33, 34, 35]. TRNGs leverage dynamic fluctuations in physical properties—such as thermal noise [36, 37], phase jitter [38], photonic fluctuation [39, 40], or electronic shot noise [41]—that evolve continuously over time, enabling significantly expanded entropy sources compared to static PUFs. This temporal dependency allows TRNGs to surpass the inherent information constraints of PUFs, generating truly stochastic sequences that are not limited by a finite device architecture. By maintaining high entropy across time‐dependent processes, TRNGs generate graphically secure random numbers with extreme unpredictability, enabling boundless cryptographic randomness beyond traditional finite models [42, 43, 44].

Integrating PUF‐based spatial randomness and TRNG‐based temporal randomness into a single platform represents a transformative advancement in hardware security and generative AI. By means of these two distinct sources of randomness, our dual‐randomness framework enables diverse latent vector sampling in generative adversarial networks (GANs). The structural randomness of the PUFs ensures the generation of unique and device‐specific image categories, while the temporal randomness introduced by TRNGs provides dynamic, fine‐grained control over image details, such as subtle facial expressions and micro‐movements. The core challenge in realizing such a system lies in the necessity for a co‐designed approach that spans material selection and device engineering. Different from conventional semiconductor‐based randomness sources that depend on predefined circuit‐level variations, our method strategically engineers intrinsic material heterogeneity to induce stochastic yet controllable entropy across (i) spatial (PUFs) and (ii) temporal (TRNGs) domains.

Here, as introduced above, we demonstrate a dual‐randomness security device that combines PUF‐based structure randomness with TRNG‐driven temporal variations by using mixed‐zinc gallate (ZGO) nanoclusters (NCs). This work presents a unified entropy platform that co‐localizes PUF and TRNG functionalities within a single nanomaterial system, offering simultaneous access to spatial and temporal randomness in a compact architecture. Our approach induces morphological variations of mixed‐ZGO NCs, causing a 10 × 20 array to exhibit diverse current variation within a 10^4^‐scale range at −1 V. This impacts the imaginary component of impedance, capacitance, and noise fluctuations, generating unique PUF identifiers. The PUF functionality achieves an inter‐cell Hamming distance (HD) of 46.21%, inter‐device HD of 50.40%, intra‐HD of 0.20%, and an average entropy of 0.83, while the TRNG functionality exhibits a uniformity of 49.89%, an inter‐HD of 51.33%, an average entropy of 0.91, and a bit‐aliasing of 49.45%, with device‐derived bitstreams from 20 independent devices meeting all 11 reported NIST criteria. Electrical analysis confirms spatial PUF randomness and temporal TRNG randomness, while long‐term measurements demonstrate stability over 91 days for the PUF and 237 days for the TRNG. To evaluate latent‐space controllability, we performed two separate demonstrations: a two‐dimensional MNIST proof‐of‐concept using a simple GAN and a 512‐dimensional animal‐image inference using a pretrained StyleGAN2‐AFHQv2 generator. In both demonstrations, the mixed‐ZGO measurements supplied hardware‐derived latent inputs, whereas image synthesis was performed by the trained or pretrained software generator. For the animal‐image demonstration, the pretrained StyleGAN2‐AFHQv2 model was used with a 512‐dimensional latent input. Each animal category—including cats, dogs, foxes, lions, and wolves—from the pretrained StyleGAN2‐AFHQv2 occupies a distinct region within the latent space [45, 46]. Based on the proposed dual‐randomness security device, where each device provides a unique current output, we can selectively sample latent vectors from precise latent‐space regions associated with desired animal categories, achieving targeted generation previously unattainable with purely random sampling approaches. The integration of temporal stochastic noise introduces subtle intra‐category variations, such as realistic shifts in facial expressions, eye positions, and fur textures. By synergistically combining PUF characteristics with TRNG noise into a unified ZGO NC‐based security platform, our approach effectively addresses the longstanding challenge in GAN‐driven imagery—simultaneously enabling deterministic category selection and fine‐grained visual diversity, significantly advancing the realism, security, and controllability of generative technologies. The integration of two distinct entropy sources within a single platform provides a hardware‐derived latent‐modulation interface for pretrained generative models.

## 2 Results and Discussion

Based on the stochastic properties of the mixed‐ZGO NCs, two functionalities can be realized: PUF and TRNG (Figure 1a). The device‐to‐device variation in the array composed of mixed‐ZGO NCs leads to different current levels across cells depending on their location, which can be utilized to implement PUFs. This property means that different sizes of random numbers can be generated in parallel for each zone of the device. On the other hand, transient current changes show stochastic behavior in which the current continuously fluctuates over time. Defects and material imperfections in the device capture and emit carriers, resulting in stochastic output currents [47, 48]. This time‐domain stochastic behavior can be utilized for TRNG applications. The measured device‐current traces are converted into hardware‐derived latent inputs and supplied to a pretrained image generator, which performs the image‐synthesis computation. The mixed‐ZGO NCs devices can use multiple input sources (position‐specific multi‐random noise) that can be classified. Using the position‐dependent current traces as hardware‐derived latent inputs, the pretrained generator synthesized multiple animal images. Each type of animal image is transformed into a variety of different appearances. Accordingly, the demonstrated application should be interpreted as hardware‐derived latent‐vector modulation for pretrained image synthesis rather than direct image generation by the mixed‐ZGO device.

**FIGURE 1:**
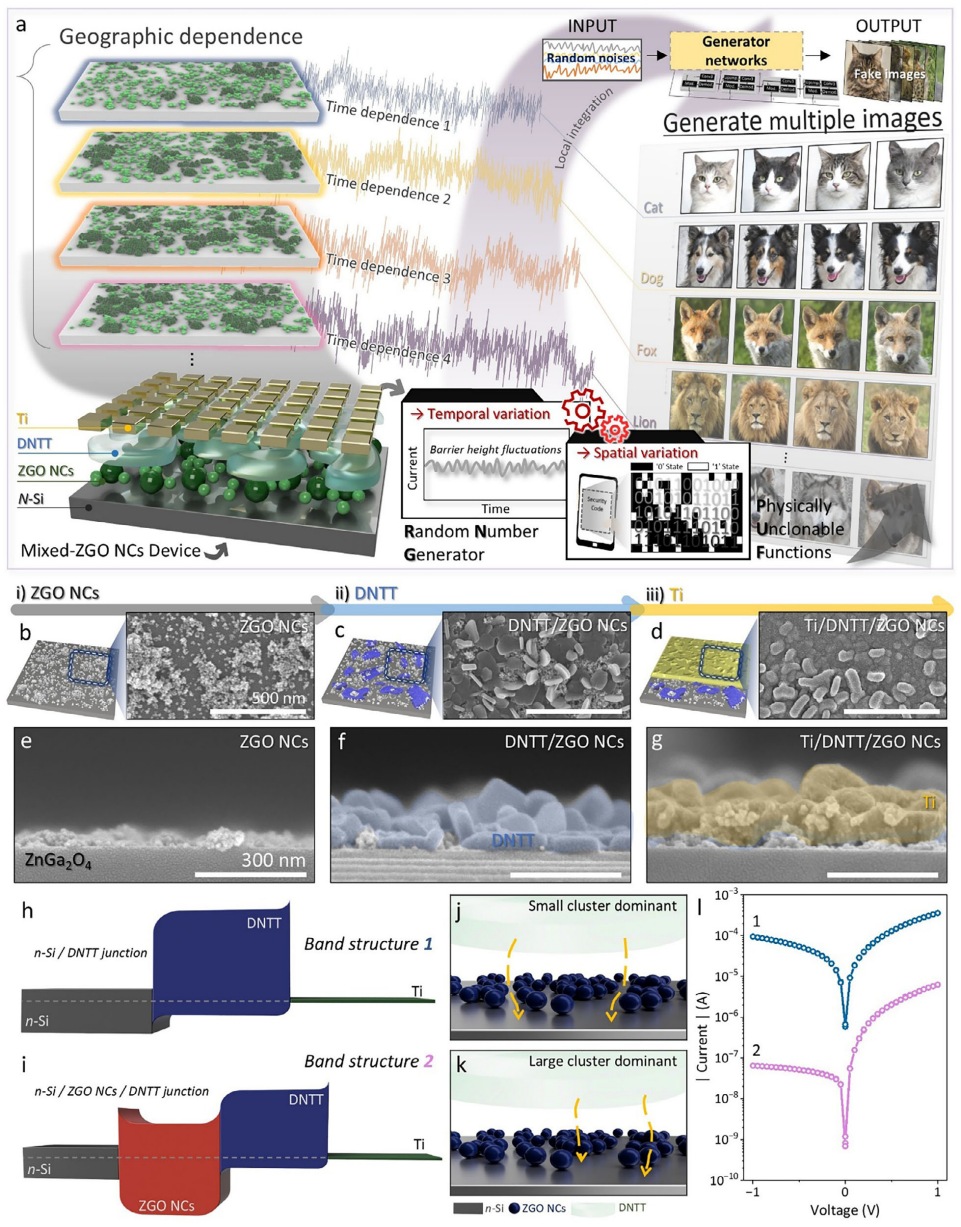
(a) Conceptual workflow in which mixed‐ZGO device measurements provide hardware‐derived latent inputs for a pretrained StyleGAN2‐AFHQv2 generator. SEM top view image including fabrication schematics of (b) ZGO NCs, (c) DNTT, and (d) Ti, respectively. SEM cross‐sectional images of (e) ZGO NCs, (f) DNTT/ZGO NCs, and (g) Ti/DNTT/ZGO NCs films. Band structure diagrams of (h) *n*‐Si/DNTT junctions and (i) *n*‐Si/ZGO NCs/DNTT junctions. Schematic of charge transfer when (j) small ZGO NCs and (k) large ZGO NCs dominate. (l) *I‐V* curves based on 1 and 2 band structures.

The device fabrication process is as follows: The ZGO NCs synthesized with different reaction times were mixed and then spin‐coated onto *n*‐Si (Figure 1b). The mixed‐ZGO NCs deposited on the *n*‐Si are randomly distributed, forming a rough surface that partially exposes the substrate. After deposition of dinaphtho[2,3‐b:2', 3'‐f]thieno[3,2‐b]thiophene (DNTT), the DNTT film covers the *n*‐Si and ZGO NCs as shown in Figure 1c. Then, the top electrode Ti was deposited (Figure 1d). The scanning electron microscope (SEM) images of the sequentially formed ZGO NCs, DNTT, and Ti clearly demonstrate the morphology and grain size differences of each material. These differences are also indirectly reflected in the elemental distributions on the film surface. The elemental distribution of each material in the film stacked with all materials was confirmed using energy‐dispersive X‐ray spectroscopy (EDS) mapping (Figure S1). Overall, the ‘Ti’ is densely distributed on the surface. In contrast, the ‘Zn’ and ‘Ga’ elements in the mixed‐ZGO NCs do not completely cover the surface and exhibit low density. Similarly, the ‘S’ elements in the DNTT exhibit a low distribution. These surface distribution characteristics are also observed in the SEM cross‐sectional images. As shown in Figure 1e, mixed‐ZGO NCs did not completely cover some *n*‐Si surfaces. On the other hand, DNTT covers the entire ZGO NCs without exposing the *n*‐Si surface (Figure 1f). The surface of devices fabricated through Ti deposition remains rough in Figure 1g. The surface characteristics of such device films can cause irregular electrical characteristics for the following two reasons. The rough surface causes differences in carrier transport distance in the vertical direction, which increases carrier recombination. Second, as mentioned above, the device undergoes two conditions in which the DNTT covers the mixed‐ZGO NCs, causing depletion width differences and affecting the electrical properties.

To characterize the proposed mixed‐ZGO NCs device, the energy level was calculated from ultraviolet–visible (UV–vis) spectroscopy and ultraviolet photoelectron spectroscopy (UPS) measurements of DNTT and mixed‐ZGO NCs (Figure S2). Mixed‐ZGO NCs exhibit strong light absorption below 250 nm and DNTT below 500 nm. The band‐gap energy (*E*_g_) of 4.88 eV (mixed‐ZGO NCs) and 2.62 eV (DNTT) were extracted using the Tauc plot. The energy level of the DNTT was identified from UPS measurements. The secondary electron cutoff (*E*_Cut‐off_) of DNTT is 16.96 eV, and the energy gap between the Fermi level (*E*_F_) and the valence band maximum (*E*_V,Max_) is 0.72 eV. The energy level information of *n*‐Si, mixed‐ZGO NCs, and titanium (Ti) was extracted from previously reported studies [49, 50, 51]. The overall energy band diagram of the device is presented in Figure S3. The work functions of *n*‐Si (cathode) and mixed‐ZGO NCs are close to their conduction bands, exhibiting *n*‐type characteristics, while the work function (*W*_F_) of DNTT is close to its highest occupied molecular orbital (HOMO) level, indicating *p*‐type characteristics. Ti (anode) is deposited on DNTT, and the negligible difference in *W*_F_ between the two materials suggests an ohmic contact.

As demonstrated in Figure 1b,e, DNTT was classified into two distinct junctions due to the partial deposition of mixed‐ZGO NCs. When the electron‐rich *n*‐Si contacts the hole‐rich DNTT, carrier diffusion occurs due to the difference in the majority carrier concentration between the semiconductors, resulting in a *p*‐*n* junction with a depletion region as illustrated in band structure 1 in Figure 1h. In band structure 2, with mixed‐ZGO NCs presented between *n*‐Si and DNTT, a *W*_F_ difference at the *n*‐*n* junction of the mixed‐ZGO NCs/*n*‐Si interface leads electrons from mixed‐ZGO NCs to diffuse into *n*‐Si, forming a Schottky junction (Figure 1i). Therefore, the ZGO NCs of small or large scale scattered on the *n*‐Si form different junctions. This causes different carrier transport paths, which in turn results in different conductance differences by device location (Figure 1j,k). At the DNTT/mixed‐ZGO NCs interface, a *p*‐*n* junction forms through the diffusion of majority carriers from each semiconductor, corresponding to band structure 1. Compared to band structure 1, band structure 2 exhibits an overall reduction in electrical conductivity with a particularly notable improvement in rectification characteristics (Figure 1l). In band structure 1, a depletion region at the DNTT/*n*‐Si interface expands under reverse bias, resulting in rectification. However, a small valence band difference of 0.19 eV facilitates leakage current, leading to poor rectifying performance (Figure S4a). In band structure 2, the wide band gap of mixed‐ZGO NCs (4.88 eV) offers a significant energy barrier of 2.21 eV at the DNTT/mixed‐ZGO NCs interface, effectively blocking holes and greatly enhancing rectification under reverse bias (Figure S4b). Additionally, the Schottky barrier at the mixed‐ZGO NCs/*n*‐Si interface contributes to lower conductance in forward bias compared to band structure 1. Based on these characteristics, the device exhibits device‐to‐device variations due to locally dominant junctions that are irregularly formed by the morphological characteristics of the mixed‐ZGO NCs, leading to variations in electrical conductivity.

The reaction time difference revealed that the synthesized ZGO NCs exhibited small and large NCs (Figure 2a,b), which were demonstrated by X‐ray diffraction (XRD) (Figure S5). Each of the synthesized ZGO NCs show peaks at 30.3° (220), 35.7° (311), 43.4° (400), 53.8° (422), 57.4° (511), 63.0° (440), 71.5° (620), and 74.5° (533) positions (JCPDF:38‐1240), which all correspond to the spinel type of ZGO. The small‐ZGO NCs contain an impurity peak corresponding to zinc hydroxide (Zn(OH)_2_), but they are negligibly weak compared to the peaks of ZGO. This phenomenon can be explained by the thermal decomposition of urea during the synthesis of ZGO NCs, which follows the reaction [52]:

(1) $$CO{\left(NH_{2}\right)}_{2}+3H_{2}O\;\to \;CO_{2}+2OH^{-}+2NH_{4}^{+}$$

**FIGURE 2:**
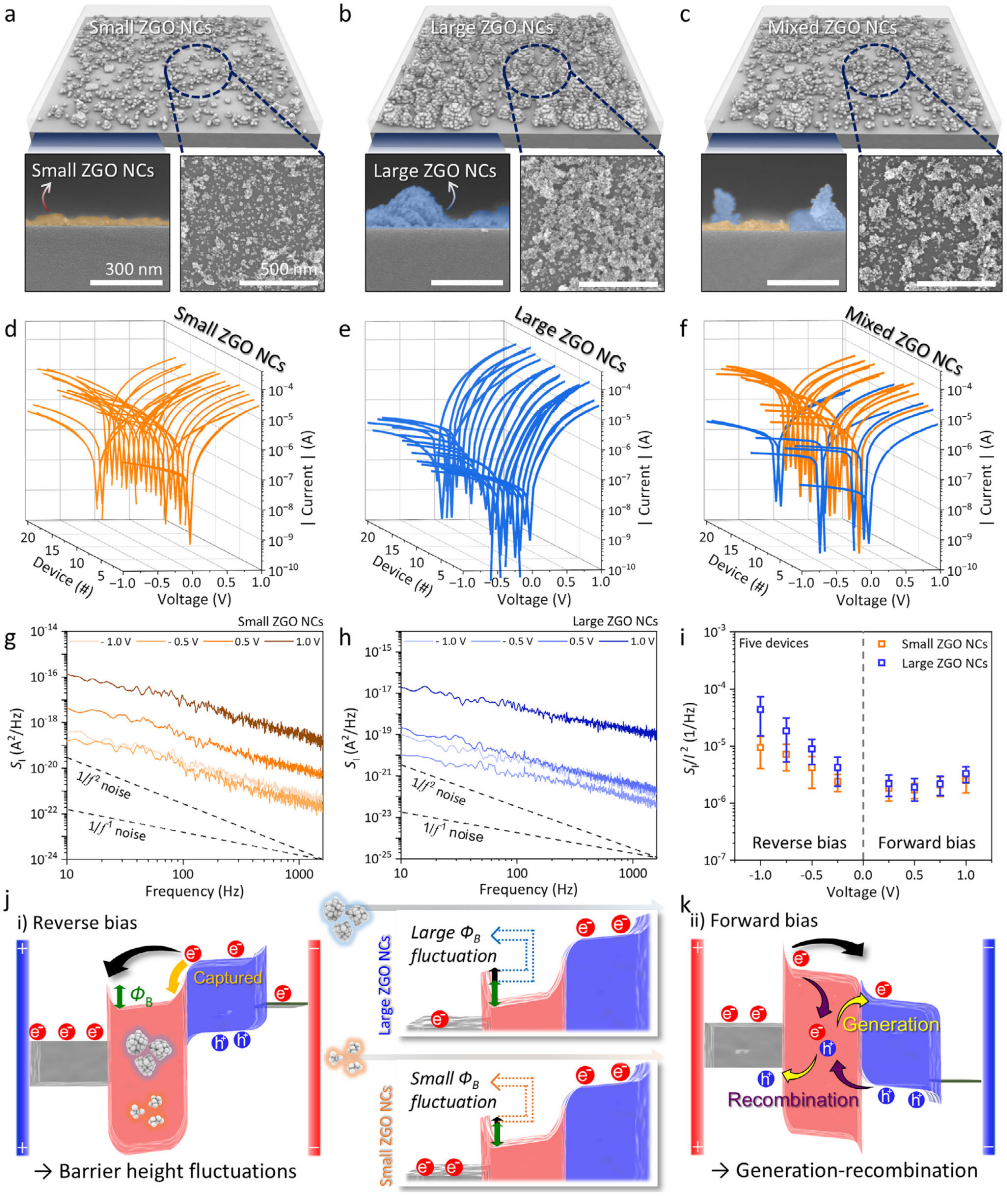
Cross‐section and top‐view SEM images of (a) small, (b) large, and (c) mixed ZGO NCs. *I–V* curves of (d) small, (e) large, and (f) mixed ZGO NCs devices. *S*_I_ versus frequency of (g) small and (h) large ZGO NCs devices. (i) Current‐normalized *S*_I_ measured on five devices, respectively (sampled at 10 Hz). Large and small ZGO NCs device mechanisms in the (j) reverse bias and (k) forward bias.

The generated hydroxyl (OH^−^) ions bond with Zn^2+^ to form Zn(OH)_2_. Due to insufficient reaction time, a small amount of Zn(OH)_2_ remains in the small ZGO NCs, but these peaks are negligibly weak compared to those of ZGO. The synthesized ZGO nanoparticles are covered with OH^−^ groups [53], which are precipitated from ${\mathrm{NH}}_{4}^{+}$ ions generated by the thermal decomposition of urea [52]. With longer reaction times, the concentration of ${\mathrm{NH}}_{4}^{+}$ ions increases, which attracts more hydroxyl‐covered ZGO nanoparticles and facilitates the formation of larger ZGO NCs (Figure S6). To investigate the size of ZGO NCs, we performed additional measurements of the chemical characteristics. As shown in Figure S7a,b, the measured pH increases from 8.84 to 9.18 under different reaction times, which is consistent with the increased concentration of ${\mathrm{NH}}_{4}^{+}$ ions described above. To further clarify the influence of reaction time on the surface charge of ZGO NCs, the Zeta potentials of the dispersions of small and large ZGO NCs were measured (Figure S7c). The small ZGO NCs exhibit a limited amount of positively charged ${\mathrm{NH}}_{4}^{+}$ ions relative to the surface OH^−^ groups on ZGO nanoparticles, which accounts for their slightly negative electrostatic potential of −6.65 mV. In contrast, the large ZGO NCs are sufficiently compensated by abundant positively charged ${\mathrm{NH}}_{4}^{+}$ ions for the surface OH^−^ groups, resulting in a positive potential of +27.99 mV. Dynamic light scattering (DLS) analysis was performed to investigate the effect of ${\mathrm{NH}}_{4}^{+}$ concentration on particle size distribution (Figure S7d). The average particle sizes (± standard deviation) of the small and large ZGO NCs were confirmed to be 137.5 ± 36.8 and 178.6 ± 36.3 nm, respectively, demonstrating that the NC size can be effectively tuned by the concentration of ${\mathrm{NH}}_{4}^{+}$ ions during the synthesis process. The size‐dependent conditions of their ZGO NCs induce morphological differences when deposited on *n*‐Si. The deposition of small‐ZGO NCs forms a relatively uniform thickness formation and exposes a large area of *n*‐Si surface (Figure 2a). In contrast, large‐ZGO NCs are more densely aggregated with ZGO NCs, resulting in a relatively smaller exposed area of *n*‐Si surface compared to small‐ZGO NCs and exhibiting inhomogeneous thickness (Figure 2b). When each ZGO NCs are mixed at a 1:1 ratio and deposited onto the *n*‐Si surface, the morphological characteristics of the two ZGO NCs are reflected and result in the irregular exposure of the *n*‐Si surface (Figure 2c). These results are also reflected in the roughness of the surface. Figure S8 shows atomic force microscopy (AFM) mapping images of surface roughness under different ZGO NCs conditions. Similar to the SEM cross‐section image, the small‐ZGO NCs exhibit the lowest roughness. Meanwhile, when the large‐ZGO NCs and mixed‐ZGO NCs are deposited, the surface is rougher compared to the small‐ZGO NCs device (root‐mean square average roughness: 13.2 nm (small‐ZGO NCs), 17.5 nm (large‐ZGO NCs), and 16.4 nm (mixed‐ZGO NCs). When comparing the surface roughness mapping images of the large‐ZGO NCs and mixed‐ZGO NCs, it can be observed that the mixed‐ZGO NCs have relatively more regions with lower roughness locally. The morphological differences caused by the formation conditions of ZGO NCs influence the junctions between materials, leading to various electrical characteristics. Devices based on small‐ZGO NCs generally exhibit high current levels above 10^−5^ A (Figure 2d), whereas those based on large‐ZGO NCs show a low current level below 10^−6^ A (Figure 2e). Interestingly, the devices fabricated with a mixture of different ZGO NCs exhibit an irregular distribution of the two current levels (Figure 2f). As illustrated in Figure 1, this occurs because the mixing of small‐ and large‐ZGO NCs results in the random exposure of the *n*‐Si surface. It is due to the selective formation of DNTT/*n*‐Si junctions (band structure 1) and DNTT/ZGO NCs/*n*‐Si junctions (band structure 2). Consequently, areas with large‐ZGO NCs exhibit low currents (band structure 2 dominates), while regions with small‐ZGO NCs show high currents (band structure 1 dominates).

To elucidate the underlying physical origin for these size‐dependent electrical differences, low‐frequency noise (LFN) analysis is employed. LFN is particularly sensitive to defects within the materials and device structure, which can significantly influence the current–voltage characteristics and stochasticity [54, 55, 56]. Time‐domain current fluctuations were Fourier transformed to extract the power spectral density (PSD) of the device for LFN analysis. Figure 2g,h show the current PSD (*S*_I_) for devices incorporating small‐ and large‐ZGO NCs. Both devices exhibit 1/*f ^γ^* noise behavior, characterized by *γ* values between 1 and 2. The measured noise of devices with different ZGO NC sizes was consistent with the DC current–voltage (I–V) curves under the applied voltage conditions (Figure S9). In general, two‐terminal devices demonstrate a 1/*f* noise, which is generally attributed to carrier fluctuations in relation to defects [57]. These fluctuations can arise from various sources, including trapping and de‐trapping processes at defects, Coulombic scattering centers, generation‐recombination processes, and barrier height fluctuations at the heterojunction [55, 58]. To investigate the origin of the noise in the devices, the analysis focuses on devices exhibiting a slope of 1 because a slope greater than 2 suggests that conduction is influenced by a single defect, making it challenging to identify the general conduction mechanism and noise source. Figure 2i shows the current‐normalized *S*_I_ (*S*_I_/*I* ^2^) for the small‐ and large‐ZGO NCs sampled at 10 Hz versus bias. The device noise behavior is analyzed in forward and reverse bias regions to investigate the bias dependency.

In a *p‐n* junction diode, the current flow in the reverse bias region is determined by the Schottky barrier at the heterojunction. The current in this region can be described by the equation [59]:

(2) $$I=AA^{*}T^{2}\exp\left(-\frac{q\phi_{B}}{k_{B}T}\right)$$

where *A* is the area, *A* ^*^ is Richardson constant, *T* is temperature, *k*_B_ is the Boltzmann constant, and *Ф*_B_ is the effective Schottky barrier height for electrons. When carriers flow through the Schottky barrier, 1/*f* noise is generated due to fluctuations in the barrier height (Figure 2j). This noise occurs when electrons are randomly captured and released by trapping states near the Schottky barrier, causing changes in the interface charge and modulating the barrier height. These fluctuations result in significant variations in the current because the reverse Schottky diode current has an exponential dependence on the barrier height. The 1/*f* noise in the reverse Schottky diode is described by the following model [58]:

(3) $$S_{I}=\frac{k^{*}}{fA\;\exp\left(-\frac{qV_{\mathrm{bi}}}{k_{B}T}\right)}$$

where *V*_bi_ is the built‐in potential of the Schottky contact, and *k* ^∗^ is the fitting parameter that considers surface recombination velocity. As shown in Figure 2j, in devices dominated by large‐ZGO NCs, a significant number of electrons drifting from Ti to *n*‐Si are captured, while in devices with small‐ZGO NCs, only a few electrons are captured. As a result, the Schottky barrier height in devices with large‐ZGO NCs fluctuates more significantly than in those with small‐ZGO NCs, increasing the sensitivity of the device to barrier height variations and resulting in more pronounced current fluctuations.

In the forward bias region, generation‐recombination noise, which is characterized by a Lorentzian spectrum, is observed. This noise can be expressed as [60]:

(4) $$S_{I}=A_{N}\;\frac{\tau_{0}}{1+{\left(\omega \tau_{0}\right)}^{2}}$$

where *A*_N_ is a noise magnitude, τ_0_ is the characteristic time of the generation‐recombination process. The PSDs of the devices do not exhibit a perfect Lorentzian spectrum, which can be attributed to the presence of multiple generation‐recombination centers during the conduction process (Figure 2k). Devices with large‐ZGO NCs show a slightly higher noise magnitude in Figure 2i. However, the difference is not as significant as observed in the reverse bias region. Therefore, it can be concluded that the stochastic behavior is more pronounced and can be maximized in the reverse bias region. Based on this analysis, the PSD of 15 devices at different locations within a 10 × 20 array composed of mixed‐ZGO NCs was analyzed (Figure S10). The PSD was measured in the reverse bias region, exhibiting distinct behaviors ranging from 1/*f* noise to 1/*f* ^2^ noise depending on the location of the device. This variation highlights the stochastic behavior of the array, which arises from the spatial randomness introduced by the intrinsic stochasticity. This variation highlights the different stochastic behavior of the array, which results from the spatial randomness introduced by the intrinsic stochasticity.

The mixed‐ZGO NC device can provide not only spatial stochasticity but also temporal stochasticity. Figure 3a shows time‐domain current fluctuations measured five times from a single device. These discrete current fluctuations arise from stochastic trapping/de‐trapping processes. For visual illustration of the current‐to‐bit conversion, the measured current was divided into ‘0’ and ‘1’ states using a single threshold of 14.32 µA, producing the 30 × 30 binary map shown in Figure 3b. This single‐threshold binary representation is distinct from the quantile‐based 3‐bit encoding protocol used to generate the bitstreams for the NIST statistical evaluation. The illustrative binary codes exhibit a uniformity of 49.89%, an inter‐HD of 51.33%, an average normalized binary Shannon entropy of 0.91 for the encoded output, and a bit‐aliasing value of 49.45% (Figure S11). This output‐based Shannon entropy metric is not a raw‐source min‐entropy estimate. These values are close to their ideal values and support the temporal randomness of the device‐derived binary outputs.

**FIGURE 3:**
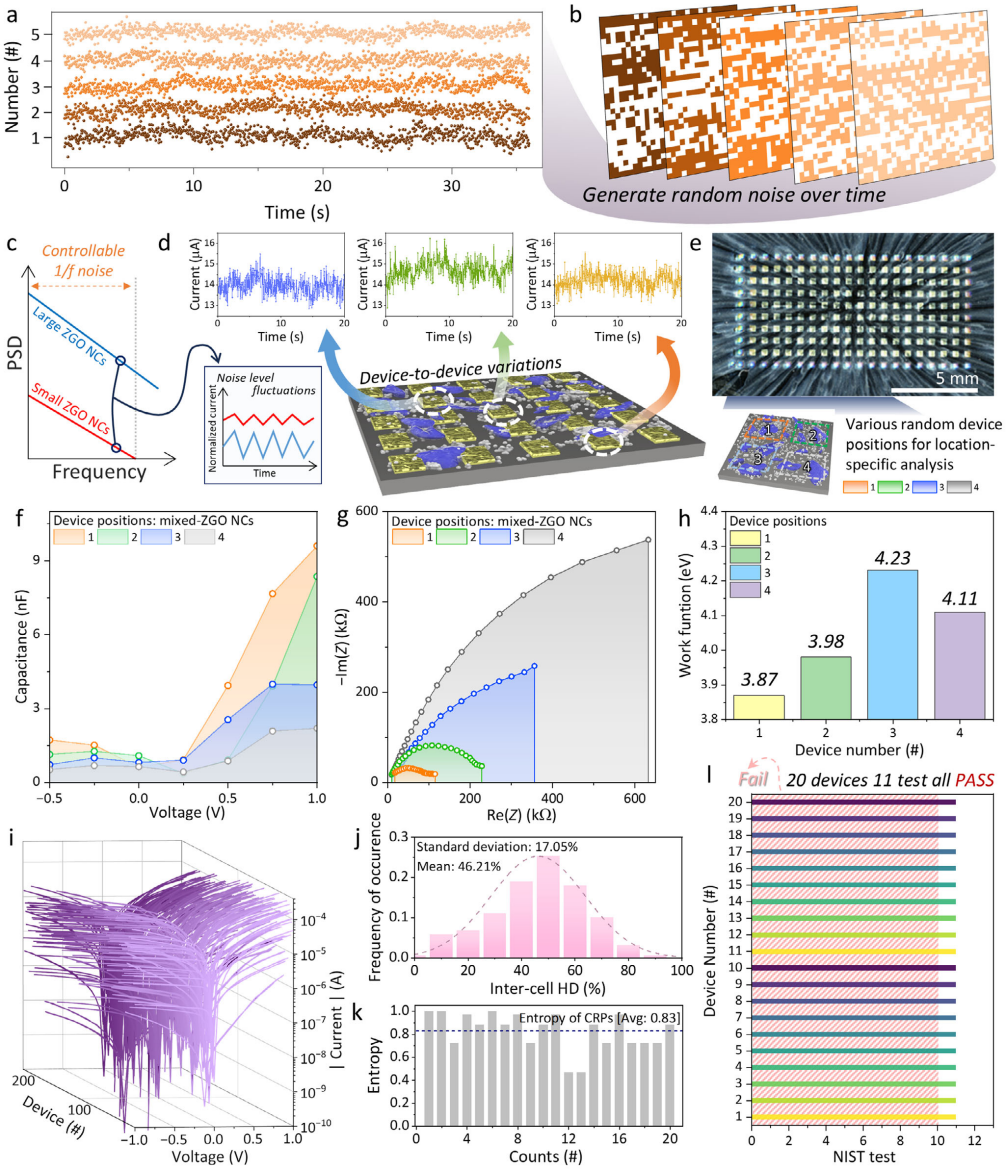
(a) Distribution of the measured noise current over time and (b) an illustrative 30 × 30 binary map generated using a single threshold of 14.32 µA. (c) Illustration of the PSD characteristics originating from the size of ZGO NCs and (d) the differences in noise current by device location. (e) Optical image of a 10 × 20 mixed‐ZGO NCs device array. Comparison of (f) capacitance, (g) impedance, and (h) *W*_F_ values measured at 4 different positions. (i) The *I–V* curve of the measured mixed‐ZGO NCs device and the (j) inter‐cell HD and (k) entropy of the bit value extracted at −1 V. (l) Number of applicable NIST tests passed by each of the 20 device‐derived bitstreams.

As shown in Figure 3c, the size dependence of the ZGO NCs influences the resistance and noise fluctuations of each device, which leads to a synergistic effect with device‐to‐device variation and time‐dependent variation (Figure 3d). Figure 3e shows optical images of 200 devices and a schematic diagram for analyzing the random physical characteristics of each device. The device resistance variation is attributed to the morphology of the mixed‐ZGO NCs, which results from differences in surface roughness [60] and depletion width [61]. In Figure 2c, the variation in rectification characteristics of each device is due to the different width of the depletion region acting as a resistance, which can be analyzed using the parallel capacitor model as follows:

(5) $$C=\frac{S\varepsilon }{w}$$

where *C* is the capacitance of the depletion region, *S* is the effective area of the device, *ε* is the dielectric constant, and *w* is the width of the depletion region. Figure S12 shows the capacitance‐voltage characteristics of the small‐ and large‐ZGO NCs devices. The large‐ZGO NCs devices exhibit capacitances ranging from 3.56 to 5.94 nF (Figure S12a), whereas the small‐ZGO NCs devices show higher values of 7.99–10.65 nF (Figure S12b). For the mixed‐ZGO NCs devices, the capacitance ranges from 2.19 to 9.60 nF, covering the ranges observed for both the small‐ and large‐ZGO NCs devices (Figure 3f). Lower capacitance suggests a wider depletion region [61, 62], which results in reduced electrical conductivity. This capacitance trend is consistent with the current‐voltage characteristics measured under alternating voltage conditions. At 1 V, the large‐ZGO NCs devices exhibit currents of 0.4–1.12 µA (Figure S13a), whereas the small‐ZGO NCs devices show higher currents of 59.6–92.8 µA (Figure S13b). The mixed‐ZGO NCs devices exhibit a broader current range of 2.01–122 µA, which includes the ranges of both the small‐ and large‐ZGO NCs devices (Figure S13c). The −Im(Z) shows an inverse trend with capacitance. The large‐ZGO NCs devices exhibit −Im(Z) of 198–387 kΩ, whereas the small‐ZGO NCs devices show lower values of 12.4–98.1 kΩ (Figures S14a,b). The mixed‐ZGO NCs devices exhibit a broad distribution of −Im(Z) that includes the ranges observed for the small‐ and large ZGO NCs devices (Figure 3g). Furthermore, the Fermi level at equilibrium depends on the junction conditions [63], as shown in Figure 1h,i. To investigate the equilibrium differences in Fermi levels depending on the location, ultraviolet photoelectron spectroscopy (UPS) was performed at four different locations. The *E*_Cut‐off_ measured at different locations shows random values of 17.33, 17.22, 16.97, and 17.09 eV, respectively (Figure S15). The *W*_F_ extracted from the UPS is lower than the DNTT (Figure 3h). This suggests that random thermal equilibrium states are generated at the interface according to the material component with which the DNTT is in contact, with devices 3 and 4 dominating for band structure 1 (device position 3: 4.23 eV and device position 4: 4.11 eV) and devices 1 and 2 dominating for band structure 2 (device position 1: 3.87 eV and device position 2: 3.98 eV). This result demonstrates that a fabricated 10 × 20 array of devices can be used as a PUF due to its inability to be artificially duplicated, with an irregular current distribution ranging from 0.01 to 94 µA at −1 V observed for 200 devices (Figure 3i). To quantitatively describe the uniqueness identification capability of PUFs, inter‐cell HD and entropy were calculated (Figure 3j,k). The extracted security code at −1 V achieves a 46.21% inter‐cell HD and an average entropy of 0.83, indicating that the security code generated from the PUF device is irregular and exhibits ideal uniqueness. To further evaluate the reliability of the PUF responses, repeated‐read measurements were performed using a 10 × 10 PUF device array. The PUF keys were extracted from the current values at −1 V over 10 repeated measurements using the same threshold of −2.59 × 10^−8^ A (Figure S16). The threshold was fixed at the median current value obtained from the first sweep. Most of the repeated measurements reproduced the reference PUF key without bit errors, and only two measurements exhibited a single‐bit mismatch. To quantify the repeated‐read reliability, the intra‐HD was calculated by comparing each PUF key obtained from the repeated measurements. Accordingly, the mean and standard deviation of the intra‐HD were 0.20% and 0.42%, respectively, corresponding to a PUF reliability of 99.8% (Figure S17). These low intra‐HD values indicate stable PUF key extraction during repeated‐read operations.

Device‐to‐device uniqueness of the PUF, 10 independently fabricated PUF devices were characterized. Figure S18a shows the pairwise inter‐device HD values among the 10 independently fabricated PUF devices. The diagonal values are 0% because they correspond to the comparison of the same PUF response, whereas the inter‐device HD values between different PUF devices are distributed close to 50%. A total of 45 unique pairwise comparisons were obtained from the 10 PUF devices. The inter‐device HD distribution shows an average value of 50.40% with a standard deviation of 4.40% (Figure S18b), indicating high device‐to‐device uniqueness of the PUF responses. Short‐range spatial correlation among PUF cells within a single array was also evaluated to characterize the randomness within the array. Figure S19a shows the spatial distribution of the current magnitude measured at −1 V across the PUF array. The current map shows no noticeable monotonic tendency to increase or decrease with position relative to neighboring PUF cells. To quantitatively characterize short‐range linear spatial autocorrelation of the PUF responses, lagged Pearson correlation coefficients were calculated using the following equation [64, 65]:

(6) $$r\left(\Delta r,\Delta c\right)=\frac{\sum_{k=1}^{n}\left(X_{k}-\bar{X}\right)\left(Y_{k}-\bar{Y}\right)}{\sqrt{\sum_{k=1}^{n}{\left(X_{k}-\bar{X}\right)}^{2}}\sqrt{\sum_{k=1}^{n}{\left(Y_{k}-\bar{Y}\right)}^{2}}}$$

where *X_k_* and *Y_k_* are the current values of the *k*th pair of PUF cells separated by the displacement vector defined by row and column lags of Δ*r* and Δ*c*, respectively, $\bar{X}$ and $\bar{Y}$ are the mean values of the two paired subsets at each spatial lag, and *n* is the number of valid paired cells at each spatial lag. For an *N_r_* × *N_c_* array, n = (*N_r_* − |Δ*r*|)(*N_c_* − Δ*c*|). A correlation coefficient close to 0 indicates weak linear correlation between spatially separated PUF cells, whereas an absolute value close to 1 indicates strong linear correlation. Figure S19b shows the two‐dimensional Pearson correlation heatmap as a function of row and column lags. At each spatial lag (Δ*r*,  Δ*c*), the correlation coefficient was calculated from PUF cell pairs separated by the corresponding spatial distance. As expected, the correlation coefficient is 1 at the 0‐lag position (0, 0), whereas low correlation coefficients are observed at spatially separated positions, supporting the absence of a pronounced short‐range linear spatial pattern in Figure S19a. The zero‐lag value represents self‐correlation and was excluded from the assessment of spatial randomness. Figure S19c shows the horizontal and vertical Pearson correlations for lags 1–4. The correlation coefficients remain close to 0 over the investigated spatial lags, indicating no detectable short‐range spatial correlation up to four cell pitches.

We re‐measured the current after 91 days to verify the long‐term stability of the proposed PUF device. As a result, a slight current decrease was observed after 91 days of air exposure (Figures S20 and S21). This decrease was observed uniformly across all devices and showed a consistent trend in the initial state shape. As shown in Figures S22a,b, the mapped PUF key based on the current obtained at −1 V after 91 days does not mismatch with the initial state, showing a bit error of 0%. The inter‐HD and entropy values ​​of the PUF key extracted after 91 days remain the same as those in the initial state, at 46.21% and 0.83, respectively (Figure S22c,d). Through this analysis, we verify the long‐term stability of PUF. In addition, we have performed tests to evaluate the environmental stability of PUF. Figure S23 shows the variation in the PUF characteristics with temperature. We measured a 4 × 4 size device under four different temperature conditions: room temperature (25°C, RT), 40°C, 60°C, and 80°C. The *I–V* curves exhibited temperature‐dependent behavior, which may be attributed to thermally activated carrier transport in silicon. Accordingly, to account for the temperature‐induced shift in the average current level, the threshold was adjusted for each condition. The detailed threshold values are provided in the Experimental Section. Figure S24 shows the PUF characteristics measured under different humidity conditions. The current showed a slight increase with increasing humidity. This increase may be associated with the adsorption of water molecules on the exposed device surface, which could modify the surface charge states and carrier transport characteristics [66]. Accordingly, the threshold was adjusted for each humidity condition to account for the humidity‐induced shift in the average current level, with the detailed threshold values provided in the Experimental Section. As a result, the 4 × 4 PUF keys remained unchanged at approximately 60%, 81%, and 90% RH, indicating that no bit errors occurred within the tested humidity range. The reliability and reproducibility of a PUF device also depend on consistent electrical operation under repeated measurements. Figure S25 shows the current‐voltage characteristics of the same PUF cell/device measured repeatedly over 1000 cycles. The device maintained stable electrical characteristics with negligible degradation throughout the repeated measurements. The current extracted at −1 V remained at a consistent level over 1000 cycles (Figure S26a), with an average current of 3.00 µA and a standard deviation of 0.13 µA, corresponding to a low coefficient of variation (CV) of 4.5% (Figure S26b). The CV was calculated as follows [67]:

(7) $$\mathrm{CV}\;=\frac{I_{SD}}{I_{average}}\;\times 100\%$$

where *I_SD_* and *I_average_* represent the average current and standard deviation, respectively, extracted at −1 V over 1000 measurement cycles. Stability under continuous bias stress was also evaluated by applying −1 V to the same PUF cell/device for 1000 s (Figure S27a). The current decreased by only 0.07 µA during the bias stress. The current‐voltage characteristics measured after bias stress remained consistent with the initial characteristics (Figure S27b). These results demonstrate stable and reproducible operation under repeated measurements and continuous bias stress.

Additionally, the time‐domain noise relevant to TRNG operation remained stable over the same period: side‐by‐side current traces at day 0 and day 237 exhibit comparable fluctuation amplitude, and temporal correlations are unchanged (Figure S28). We extracted the security code based on the noise current measured after 237 days, which exhibited an unpredictable binary pattern, as shown in Figure S29a. The calculated uniformity and inter‐HD deviate by less than 3% from the initial values (Figures S29b,c), and entropy differs by less than 1% (Figure S29d). All metrics maintain close to their ideal values, confirming stable long‐term performance. These results are attributed to the chemical stability of ZnGa_2_O_4_ [49, 68] and the high resistance of DNTT against oxidation arising from its large ionization energy [69]. The environmental stability of the physical entropy source was further evaluated using one representative mixed‐ZGO device under ambient conditions (RT, 760 Torr), vacuum conditions of approximately 20 Torr at RT, and a high‐temperature condition at 60°C (Figure S30). Although the magnitude of the normalized current‐noise spectra, *S*_I_/*I* ^2^, differed across the three conditions, particularly at 60°C, they retained a similar 1/*f* frequency dependence at −1, −0.5, 0.5, and 1 V (Figure S30a–c). These similar trends in the noise characteristics indicate that the stochastic current fluctuations underlying the TRNG output maintain their characteristic behavior under varying environmental conditions. The 30 × 30 TRNG binary maps generated from the time‐domain current fluctuations measured at −1 V exhibited stochastic binary patterns under all three conditions (Figure S30d–f). The corresponding security parameters also remained close to their ideal values, with uniformity ranging from 49.44% to 51.05%, inter‐HD from 49.82% to 50.17%, and entropy from 0.91 to 0.96 (Table S1). These results support stable TRNG operation under the tested environmental conditions. To evaluate interval occupancy using test data that were not used for quantile‐boundary calibration, we performed a chronological calibration‐test analysis using the device 9‐1 transient‐current traces measured at −1 V under ambient, vacuum, and 60°C conditions. The first 2222 of 22 222 samples from each trace were used exclusively to determine the calibration median and seven quantile boundaries. These parameters were subsequently frozen and applied without recalibration to the remaining 20 000 test samples. The resulting test‐set interval occupancies are presented in Figure S31. Since the seven quantile boundaries divide the calibration distribution into eight equal‐probability intervals, the ideal occupancy of each interval is 1/8, or 12.5%, provided that the statistical distribution of the test segment remains consistent with that of the calibration segment. Using the fixed calibration parameters, the test‐set occupancies across the eight quantile‐defined output intervals ranged from 9.84% to 16.38% (Ambient: 9.84% to 16.38, Vacuum: 11.07% to 14.14%, Temperature: 9.96% to 13.87%). Although some variation from the ideal occupancy of 12.5% was observed, the interval populations remained reasonably close to the expected value, indicating that the held‐out test data retained a broadly balanced distribution.

For the NIST statistical evaluation, transient‐current traces from 20 devices were sampled at 3200 samples s^−1^ and converted into bitstreams using the quantile‐based 3‐bit encoding protocol described in the Experimental Section and Supporting Information. Briefly, the absolute current fluctuation, *d*(*t*) = |*I*(*t*) – median(*I*)|, was divided into eight quantile‐defined intervals using seven trace‐specific quantile boundaries at cumulative probabilities of 1/8, 2/8, …, and 7/8. The interval labels from 0 to 7 were encoded as 000 to 111 and concatenated in chronological order. With no temporal decimation (*D* = 1), the bitstream generation rate was 9600 bits s^−1^, calculated from the sampling rate of 3200 samples s^−1^ and 3‐bit encoding assigned to each current sample. This rate is determined by the sampling and encoding procedures and represents the encoded‐bit throughput rather than the entropy generation rate of the physical noise source. Accordingly, each 60 000‐bit sequence corresponded to 20 000 current samples and 6.25 s of data. The quantile boundaries were determined before bit encoding and were not adjusted based on the NIST results. No additional filtering or post‐processing was applied. All 20 device‐derived bitstreams exceeded the acceptance threshold of 0.01 for the 11 applicable NIST criteria shown in Figures 3l and S32. The Random Excursions and Random Excursions Variant tests were excluded from the pass assessment because the available 60,000‐bit sequences did not satisfy the recommended input‐length requirements.

The proposed PUF‐TRNG dual randomness security device addresses the challenge in hardware‐based generative systems of achieving both high entropy and deterministic control in latent vector sampling. Conventional TRNGs provide the stochasticity necessary for high entropy but do not offer control over the mean of the latent vector, making it difficult to target specific regions in the latent space. In contrast, PUFs offer device‐specific deterministic signatures; however, they yield only a finite set of codes, which limits their application for continuous sampling. By integrating spatially dependent deterministic offsets that originate from locally irregular mixed‐ZGO NCs with time‐dependent TRNG noise, the proposed device resolves this dilemma. This dual randomness strategy not only preserves high entropy but also allows controlled modulation of the latent vector mean, thereby enabling selective sampling within the latent space. Two separate generative‐model workflows were evaluated: a simple GAN with a two‐dimensional latent space for the MNIST proof‐of‐concept and a pretrained StyleGAN2‐AFHQv2 model with a 512‐dimensional latent input for animal‐image inference. Figure 4a illustrates pretrained StyleGAN2‐AFHQv2 inference using a 512‐dimensional hardware‐derived latent input. In this architecture, experimentally measured current samples from two selected mixed‐ZGO device traces are normalized and assigned to the 512 coordinates of the latent vector *z*. The mixed‐ZGO devices do not perform the mapping‐ or synthesis‐network computation; the pretrained StyleGAN2‐AFHQv2 mapping and synthesis networks process *z* and perform the image‐synthesis computation. While the TRNG operation provides stochastic temporal fluctuations, it inherently produces noisy latent vectors with uncontrolled means. This poses a challenge for generating images from a specific category since the latent vector must be positioned precisely within the latent space. A two‐dimensional projection of the latent space used in the image generation process is presented as shown in Figure 4b. Where different colors indicate that each MNIST digit occupies a distinct distribution within the latent space (Figure S33). An overlay of three concentric circles on the latent space projection represents different noise regimes (Figure 4c). The smallest circle corresponds to low noise, the intermediate circle to medium noise, and the largest circle to high noise. Under low‐noise conditions, the latent vectors are confined near the center (around (0,0)), leading to homogeneous outputs, as shown in Figure S34, where low sigma values yield MNIST outputs from a single category. Conversely, high noise conditions spread the latent vectors uniformly across the latent space, resulting in diverse outputs that fail to selectively target a desired class. These observations underscore the need for a dual randomness approach that simultaneously achieves high stochasticity and controlled deterministic mean adjustments. Figure S35 shows nine candidate transient‐current traces with current values ranging from 5 to 30 µA. For each 512‐dimensional latent vector, two traces were selected, 256 consecutive samples were extracted from each trace from a common starting index *t*_0_, and each sample was linearly normalized to the range from −2 to 2. Note that we also evaluated three normalization routes: Linear, Rank (normal‐scores), and Quantile‐matched (Qmatch), under matched settings and a software‐independent and identically distributed (i.i.d.) pseudo‐random number generator (PRNG) baseline. Across the three preprocessing routes, images synthesized using hardware‐derived latent inputs showed FID, KID, and Inception‐feature‐space diversity‐proxy values broadly comparable to those obtained using the software i.i.d.‐Gaussian PRNG baseline under the evaluated conditions (Figure S36). The principal Figure 4 pipeline uses the fixed min–max mapping from the measured current range of 5–30 µA to the latent range of −2 to 2, as described above. Figure S37 presents a separate preprocessing‐sensitivity benchmark using the Linear‐standardized, Rank‐normal, and Qmatch routes. In contrast, the degraded software‐PRNG controls using the small‐state LCG4 and XOR4 generators exhibit less favorable FID and KID values and lower Inception‐feature‐space diversity‐proxy values under the evaluated mappings (Figure S37). Figure S38 presents the separate two‐dimensional MNIST proof‐of‐concept, showing how the selection of a device pair corresponding to the *Z*_1_ and *Z*_2_ coordinates affects the predominant generated digit class. Supporting Information provides a further detailed discussion of the simulation and experimental processes (Figures S33–S35 and S38).

**FIGURE 4:**
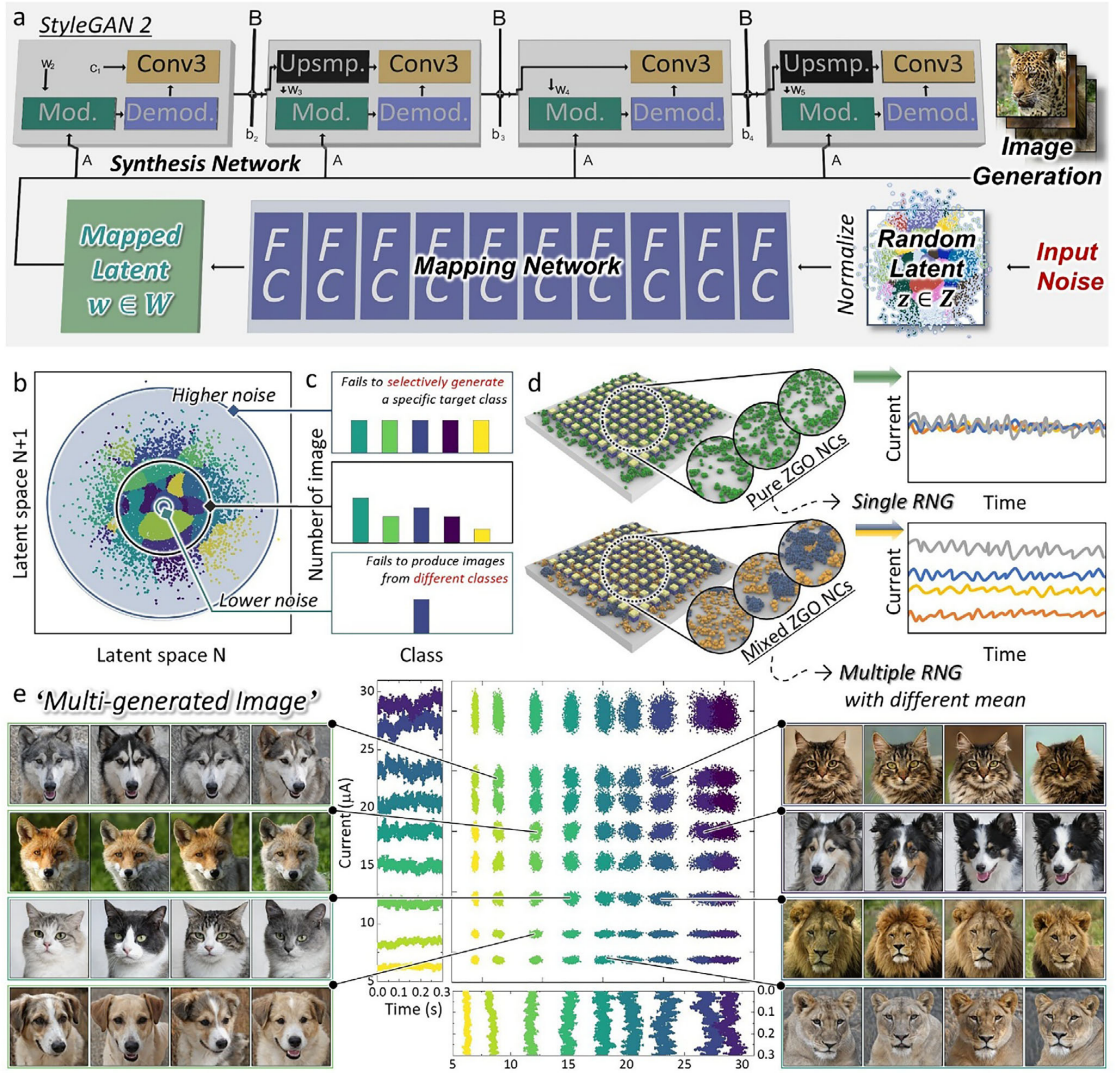
(a) Schematic of pretrained StyleGAN2‐AFHQv2 inference using a 512‐dimensional hardware‐derived latent vector. For each latent vector, two measured device traces provide 256 consecutive current samples each, which are assigned to the even‐ and odd‐indexed coordinates, respectively. The pretrained mapping and synthesis networks perform image synthesis. (b) Two‐dimensional latent‐space projection used for the separate MNIST proof‐of‐concept. (c) Overlay of concentric circles on the MNIST latent space in (b), indicating low‐, medium‐, and high‐noise regimes. Under low‐noise conditions, the latent vectors are confined near the center and produce homogeneous outputs, whereas high‐noise conditions spread the vectors across multiple classes. (d) Schematic of the mixed‐ZGO NC conditions that produce spatially dependent current offsets across the device array. (e) Animal images synthesized by the pretrained StyleGAN2‐AFHQv2 generator using hardware‐derived latent inputs. The mixed‐ZGO devices provide the measured latent‐input values and do not perform the generator computation.

Figure 4d presents a schematic diagram of the proposed device architecture designed to resolve the entropy‐control trade‐off in latent sampling. The upper panel displays pure ZGO NCs forming uniform NCs, which result in nearly identical current mean values. In contrast, the lower panel illustrates the proposed mixed‐ZGO NCs configuration, where regional variations in NCs size produce multiple TRNG outputs with distinct mean offsets. This irregular morphology of mixed‐ZGO NCs allows selective targeting of specific latent‐space coordinates, effectively controlling the latent vector mean while simultaneously preserving high stochastic entropy derived from the TRNG functionality. Using hardware‐derived latent vectors constructed from the measured mixed‐ZGO current traces, animal images were synthesized by the pretrained StyleGAN2‐AFHQv2 generator, including cats, dogs, foxes, lions, and wolves (Figure 4e) [45, 46]. Transient‐current traces from nine distinct devices were available as candidate hardware sources. For each 512‐dimensional latent vector, two traces were selected from these nine candidate traces. To examine the coupling between the device‐dependent mean‐current component and the temporal‐fluctuation component, we implemented co‐localized and de‐localized pairing controls. Each measured trace was decomposed as *I*_i_​(*t*) = *µ*_i_​ + *δI*_i_​(*t*), where is the mean‐current component and *δI*_i_​(*t*) = *I*_i_​(*t*) − *µ*_i_ is the temporal‐fluctuation component. In the co‐localized condition, both components were obtained from the same device trace, *I*_i_^co^​(*t*) = *µ*_i_​ + *δI*_i_​(*t*). In the de‐localized condition, the mean‐current component from one device was combined with the fluctuation component from another device, *I*_i,π(i)_^de^​(*t*) = *µ*_i_​ + *δI*_π(i)_​(*t*), where *π*(*i*) ≠ *i*. Figure S39 compares FID, KID, and the Inception‐feature‐space diversity proxy for the co‐localized and de‐localized protocols, showing broadly similar values under the matched conditions. Figure S40 compares the mean current with the lag‐1 autocorrelation and normalized standard deviation. Together with the similar metrics obtained for the two pairing protocols, these weak correlations indicate weak coupling between the mean‐current and temporal‐fluctuation components under the evaluated conditions, rather than strict statistical independence. The measured transient currents, which capture the inherent stochastic noise of the device, are normalized using the function:

(8) $$Z_{1},\;Z_{2}=\frac{I-17.5\;\mu A}{6.25\;\mu A}$$

scaling the *I* value (ranging from 5 to 30 µA) to a range between −2 and 2. From each selected device trace, 256 consecutive current samples were extracted from the same starting index *t*_0_. The normalized samples from the first trace were assigned to the even‐indexed latent coordinates, and those from the second trace were assigned to the odd‐indexed coordinates according to:

(9) $$z\left[2k\right]=\mathrm{Normalize}\left(I_{a}\left[t_{0}+k\right]\right)$$

(10) $$z\left[2k+1\right]=\mathrm{Normalize}\left(I_{b}\left[t_{0}+k\right]\right)$$

where *k* = 0, 1, …, 255. Thus, all 512 latent coordinates were populated by measured temporal‐current samples, comprising 256 samples from each of two selected device traces. No scalar *Z*_1_ or *Z*_2_ value was repeated, and no coordinate was filled using a software‐generated random value; only the normalization and even/odd coordinate assignment were deterministic. The resulting latent vector was reshaped to [1, 512] and supplied to *G*_ema_ of the pretrained StyleGAN2‐AFHQv2 model (stylegan2‐afhqv2‐512 × 512.pkl) using *truncation_psi* = 0.7 and *noise_mode* = *const*. The pretrained generator was used only for inference, without retraining or fine‐tuning. Beyond the default *ψ* = 0.7, we performed a standard truncation sweep (*ψ* = 0.4–1.0) to probe the fidelity–diversity trade‐off in StyleGAN2‐AFHQv2. Figures S41–S43 report the truncation sweep for FID, KID, and the Inception‐feature‐space diversity proxy, respectively. Across *ψ* = 0.4−1.0, the hardware‐derived and software i.i.d.‐Gaussian PRNG latent inputs exhibited broadly comparable metric trends under matched conditions. Although all generated images fall within the same target animal category, they exhibit subtle variations in posture, texture, and feature details. To benchmark hardware‐derived latent inputs against the established software practice, we also generated i.i.d. standard‐normal latents in software (PRNG baseline) and compared them under matched sample counts, truncation, and reference sets. Figure S36 summarizes FID, KID, and the Inception‐feature‐space diversity proxy across the Linear, Rank, and Qmatch preprocessing routes, whereas Figure S44 presents the PCA comparison of hardware‐derived and software‐PRNG latent inputs. Under the evaluated conditions, the two input types show broadly comparable image‐quality metrics and latent‐space distributions. This outcome confirms that the dual randomness strategy, which combines the deterministic offsets from the irregular NCs with time‐dependent TRNG noise, effectively guides the synthesis network to generate images that are both category‐consistent and rich in intra‐class diversity.

Recent studies have reported various approaches for integrating PUF and TRNG function within a single hardware platform by exploiting different forms of intrinsic device randomness. Generally, PUFs primarily rely on static device‐to‐device variations arising from fabrication processes or structural randomness, whereas TRNGs utilize dynamic stochastic variations, such as temporal or cycle‐to‐cycle fluctuations within individual devices. Accordingly, various integration strategies have been explored, including SRAM arrays, circuit‐level integration, vertically integrated devices, and memristor arrays. In this study, the mixed‐ZGO NC platform implements both functions using the same simple two‐terminal device architecture. Current variations arising from the spatial distribution of ZGO NCs are utilized for PUF operation, while temporal current fluctuations serve as the entropy source for TRNG operation. This approach enables the extraction of both static and dynamic randomness without requiring additional device integration for the two functions. Furthermore, multiple devices with distinct fluctuation characteristics can be utilized as independent TRNG sources, enabling the demonstration of a multi‐source TRNG application. Table S2 compares recently reported integrated PUF‐TRNG studies based on key security parameters, including uniformity, inter‐HD, entropy, long‐term stability, and NIST results. PUFs and TRNGs require distinct entropy characteristics, which has prompted several studies to employ additional readout circuits capable of simultaneously extracting both metrics [70, 71]. In comparison, our work realizes a combined PUF‐TRNG within a single platform by exploiting the intrinsic entropy sources of the device, without resorting to complex circuit configurations. Furthermore, the PUF and TRNG were evaluated under various environmental conditions, including long‐term stability, thermal stability, and humidity variation for the PUF, and temperature variation and vacuum conditions for the TRNG, while successfully maintaining randomness (Figure S45). We believe that these results highlight the strength and distinctiveness of our work.

## 3 Conclusion

In summary, we present a dual‐randomness security device that integrates PUFs and TRNGs using mixed ZGO nanoclusters. The distribution of large and small NCs induces spatial variations in electrical properties, enabling device‐to‐device differences in conductance and thus PUF functionality. Measurements across a 10 × 20 array reveal clear spatial variation, supported by capacitance, impedance, and work function analyses at multiple locations. These spatial features yield an inter‐cell HD of 46.21%, inter‐device HD of 50.40%, intra‐HD of 0.20%, and an average entropy of 0.83. Temporal current fluctuations provide the basis for TRNG operation. The single‐threshold binary mapping used for the illustrative randomness analysis yields a uniformity of 49.89%, an inter‐HD of 51.33%, an average entropy of 0.91, and a bit‐aliasing value of 49.45%. Separately, quantile‐encoded 60 000‐bit sequences from 20 devices exceeded the acceptance threshold of 0.01 for all 11 reported NIST criteria without additional filtering or post‐processing. The reported statistical tests and normalized binary Shannon entropy characterize the encoded output but do not constitute a raw‐source min‐entropy assessment or certification of cryptographic suitability. The mixed‐ZGO measurements provide spatial offsets and temporal fluctuations for stochastic latent‐vector modulation. The MNIST study serves as a separate two‐dimensional proof‐of‐concept, while the animal images were generated by a pretrained StyleGAN2‐AFHQv2 model using 512‐dimensional latent vectors constructed from two measured device traces. Thus, the device performs hardware‐derived latent modulation rather than direct image generation.

## 4 Experimental Section/Methods

### 4.1 Materials

Zinc acetate dihydrate (Zn(CH_3_COOH)_2_∙2H_2_O, ≥98%), gallium nitrate hydrate (Ga(NO_3_)_3_∙xH_2_O, 99.9%) and urea (CH_4_N_2_O, ≥98%), chloroform (CHCl_3_, ≥99%), DNTT (C_22_H_12_S_2_, >99%), were purchased by Sigma–Aldrich (USA). Ethanol (C_2_H_6_O, anhydrous, 99.8%) was supplied by Alfa Aesar (South Korea). 2‐propanol (C_3_H_8_O, ≥99.5%) was obtained from DUKSAN (South Korea). Titanium pellet (Ti, 99.995%) was purchased from iTASCO (South Korea).

### 4.2 Synthesis of Small‐ and Large‐ZGO NCs

Size variation ZGO NCs were synthesized using a one‐pot hydrothermal process. 0.05 mol zinc acetate dihydrate and 0.1 mol gallium nitrate hydrate were dissolved in 30 mL of deionized water. The mixture was stirred until homogeneous and then added to 6 g of urea. The solution was stirred for 10 min and transferred to a glass bottle with a cap. The size of the ZGO NCs depends on the reaction time at 90°C. When the solution is reacted for 24 h, smaller ZGO NCs are formed, whereas a 36 h reaction results in larger ZGO NCs. After cooling to room temperature, the ZGO NCs solution was collected and washed with 2‐propanol via centrifugation.

### 4.3 Preparation of Coating Solutions

Small‐ and large‐ZGO NCs were dispersed in a co‐solvent of chloroform and ethanol in a 3:1 volume ratio with a concentration of 20 mg∙mL^−1^. Mixed‐ZGO NCs solution was prepared by mixing the small‐ and large‐ZGO NCs solution in a 1:1 volume ratio.

### 4.4 Fabrication Process

We prepared *n*‐doped Si substrates. *n*‐Si substrates cleaning was ultrasonicated in ethanol for 10 min and in 2‐propanol for 10 min, and then dried on a hot plate at 100°C. The synthesized small‐ZGO NCs, large‐ZGO NCs, and mixed‐ZGO NCs were spin‐coated. It was spin‐coated for 30 s under the conditions of 3000 rpm and annealed at 120°C for 1 h. After that, DNTT of 20 nm was deposited at a rate of 0.2–0.4 Å∙s^−1^ through a thermal evaporator. Finally, the top electrode Ti was formed to a thickness of 70 nm through the e‐beam evaporator.

### 4.5 Characterization

The *I–V* characteristics of ZGO NCs device were obtained using a Keithley 4200 semiconductor parameter analyzer at room temperature. The ambient RH was monitored using a commercially available digital thermo‐hygrometer (CAS, T023, Korea). The top view and cross‐sectional images of the device for comparison by ZGO NCs size were analyzed using SEM (S‐4700, Hitachi). The crystal structures and size of the small and large ZGO NCs synthesized were analyzed using XRD (SmartLab, Rigaku) and DLS (ELS‐8000, Otsuka Electronics, Osaka, Japan). The elemental distribution of the film is observed through EDS mapping. pH of solution synthesized small and large ZGO NCS were meawured with pH meter (ST3100‐F, OHAUS, New Jersey, USA). Zeta potentials of the dispersions of small and large ZGO NCs were characterized by Zeta potential analyzer (ELS‐8000, Otsuka Electronics, Osaka, Japan). The roughness of ZGO NCs of different sizes was confirmed using AFM (Park NX10, Park Systems). ZGO and DNTT were measured for their optical bandgaps using UV–vis spectrophotometry (Lambda 750, PerkinElmer) to determine the absorbance at different wavelengths and using the Tauc plot. The HOMO and LUMO levels of DNTT were obtained by calculating the *E*_Cut‐off_ and *E*_V,Max_ values gained from UPS (AXIS SUPRA, Kratos, Inc.) analysis.

### 4.6 LFN Analysis

LFN characteristics of two‐terminal ZGO NCs‐based devices were systematically analyzed according to NCs size. Noise measurements were conducted using a low‐noise current preamplifier (SR570) connected to a dynamic signal analyzer (Agilent 35670A) to accurately capture and characterize LFN signals from the samples. Note that bias voltage is also applied using SR570A. Each sample, differentiated by NCs size, was individually measured under identical conditions to determine size‐dependent noise behavior by varying the read biases. For samples containing mixed NCs sizes, LFN measurements were performed at multiple localized positions. This allowed for detailed mapping of defect distributions across different areas of the sample. Such positional noise analysis provided insight into the spatial heterogeneity of defects related to NCs size variation within the ZGO films.

### 4.7 PUF Bit‐Generation Procedure

PUF bit values of ‘0’ and ‘1’ were divided based on the current values extracted at −1 V. The threshold was arbitrarily set to the median current value at which the ratio of ‘0’ and ‘1’ in the bitstream was 50%. In the case of 10 repeated measurement data, the same threshold value (−2.59 × 10^−8^ A) was applied to all 10 sets of bit values. To account for the temperature‐induced shift in the average current level, the threshold was adjusted for each temperature condition (RT: −7.55 × 10^−8^ A, 40°C: −2.02 × 10^−7^ A, 60°C: −2.75 × 10^−7^ A, and 80°C: −4.99 × 10^−7^ A). To account for the humidity‐induced shift in the average current level, the threshold was adjusted for each humidity condition (60% RH: −2.26 × 10^−7^ A, 81% RH: −2.30 × 10^−7^ A, and 90% RH: −2.35 × 10^−7^ A).

### 4.8 TRNG Bit‐Generation Procedure

Transient‐current traces were sampled at 3200 samples s^−1^. For the original trace‐specific encoding, the median current of each trace I(t) was calculated, and the absolute fluctuation amplitude was defined as *d*(*t*) = |*I*(*t*) – median(*I*)|. The empirical distribution of *d*(*t*) was divided into eight quantile‐defined intervals using seven quantile boundaries at cumulative probabilities of 1/8, 2/8, …, and 7/8. These quantile boundaries were calculated before bit encoding for each measured trace and were used as deterministic digitization thresholds; they were not adjusted or optimized based on the NIST test results. Each of the eight intervals was assigned an integer label from 0 to 7, and each label was converted into a 3‐bit binary word: 0 = 000, 1 = 001, …, and 7 = 111. The resulting 3‐bit words were concatenated in chronological order to generate the TRNG bitstream. Thus, each current sample was converted into a 3‐bit word. No temporal decimation was applied (*D* = 1). Accordingly, the bitstream generation rate was 9600 bit s^−1^, calculated as 3200 samples s^−1^ × 3 bits sample^−1^. This value represents the encoded‐bit throughput determined by the sampling and encoding procedures and should not be interpreted as the entropy rate of the physical noise source. A 60 000‐bit sequence corresponded to 20 000 current samples and 6.25 s of data. No additional post‐processing, including Von Neumann correction, XOR whitening or mixing, digital filtering, or error‐correction coding, was applied.

For the independent chronological calibration‐test analysis, each of the three device 9‐1 transient‐current traces containing 22 222 samples was divided before any statistical calculation. The first 2222 samples, corresponding to approximately 10% of each trace, were used exclusively for calibration. The calibration median, I_cal, med, was calculated from this segment, and the absolute calibration fluctuations, d_cal(t) = |I(t) − I_cal, med|, were used to determine seven empirical quantile boundaries at cumulative probabilities of 1/8, 2/8, …, and 7/8. The calibration median and all seven quantile boundaries were then frozen and applied without modification to the remaining 20 000 test samples. No median, threshold, or quantile boundary was recalculated using the test segment. Each test sample was assigned to one of the eight intervals using the frozen calibration parameters and encoded as a 3‐bit binary word from 000 to 111. The words were concatenated chronologically to produce a 60 000‐bit test sequence for each environmental condition. This procedure was applied to the device 9‐1 transient‐current traces measured at −1 V under ambient, vacuum, and 60°C conditions. The resulting test‐set eight‐bin occupancies are presented in Figure S31.

### 4.9 Randomness Evaluation

The randomness of the bit‐mapping code of ‘0’ and ‘1’ obtained from the PUF and TRNG functions was evaluated through uniformity, inter‐HD, entropy, and bit‐aliasing [12, 72]. The NIST bitstreams were generated separately using the quantile‐based 3‐bit protocol described in the preceding subsection.

(11) $$Uniformity\;\;\left(\%\right)=\frac{1}{n}\sum_{i=1}^{n}r_{i}\times 100$$

where *r*_i_ is the number of the ‘0’ bit, and *n* is the length of the bit‐mapping string.

(12) $$\begin{array}{ccc} Inter-HD\;\;\left(\%\right) & = & \frac{2}{k\left(k-1\right)}\sum_{i=1}^{k-1}\sum_{j=i+1}^{k}\;\frac{HD\;\left(R_{i}\;\left(n\right),R_{j}\;\left(n\right)\right)}{n}\\ & & \times \;100 \end{array}$$

where *k* is the number of bit‐mapping code*, R*_i_ (*n*) and *R*_j_ (*n*) are the *n*‐bit responses of the *i*‐th and *j*‐th.

(13) $$Bit-aliasing\;\;\left(\%\right)=\frac{1}{q}\sum_{p=1}^{q}R_{p}\left(j\right)\times 100$$

where *q* is the number of mappings. Also, the *j*‐th bit of the *n*‐bit response in the *p*‐th mapping was expressed as *R*_p_ (j).

(14) $$Entropy=-[p\log_{2}p+\left(1-p\right)\log_{2}\left(1-p\right)]$$

where *p* means the uniformity of the bit‐mapping code. The entropy metric reported in this work is the normalized binary Shannon entropy calculated from the encoded bitstream. It is distinct from the min‐entropy of the raw physical noise source and should not be interpreted as a measure of certified cryptographic suitability.

### 4.10 MNIST Latent‐Space Proof‐of‐Concept

The image generation simulation method involves mapping experimentally measured transient currents from dual‐randomness security devices onto the latent space of a trained GAN model for MNIST image synthesis. Device‐specific mean current outputs, ranging between 5 and 30 µA, are normalized to latent coordinates (*Z*_1_, *Z*_2_) via a simple linear scaling procedure, facilitating accurate representation within the GAN's latent distribution. By carefully selecting device pairs based on their deterministic offsets, it is possible to target specific latent space regions, enabling reproducible generation of desired digit categories while maintaining controlled variability in image features. Detailed procedures and analyses are provided in Supporting Information (Figure S38). Hardware‐derived latent inputs were benchmarked against a software i.i.d. standard‐normal baseline (PRNG‐iid) under matched sample counts, truncation (*ψ* = 0.7), and Linear/Rank/Qmatch mappings. For each condition, comparative FID and KID estimates were calculated from the InceptionV3 feature representations of 96 synthesized images and a matched reference set of 96 images. The reference images were generated using the same pretrained StyleGAN2‐AFHQv2 generator with independent 512‐dimensional i.i.d. standard‐normal latent vectors, z∼N(0,1), rather than being sampled directly from the AFHQv2 real‐image dataset. The generator, truncation parameter, preprocessing, sample size, and image‐synthesis settings were held identical within each comparison. Accordingly, the reported values quantify relative distributional differences from the software‐Gaussian latent baseline and should not be interpreted as conventional dataset‐level FID or KID scores against real AFHQv2 images. Under the evaluated conditions, the hardware‐derived latent inputs showed broadly comparable metric values to the PRNG‐iid baseline in Figure S36, and the corresponding latent‐space distributions were compared by PCA in Figure S44. To probe robustness, we introduced degraded, resource‐limited PRNG controls using small‐state generators (LCG‐4 and XOR‐4) under the identical mappings and references. Under the evaluated conditions, these controls yielded less favorable FID and KID values and lower Inception‐feature‐space diversity‐proxy values than the hardware‐derived and PRNG‐iid latent inputs (Figure S37).

We also performed a truncation sweep (*ψ* = 0.4–1.0) to assess the fidelity–diversity trade‐off. Figures S41–S43 report FID, KID, and the Inception‐feature‐space diversity proxy, respectively, across *ψ* = 0.4–1.0. The hardware‐derived and PRNG‐iid latent inputs showed broadly comparable metric trends under the evaluated conditions. For latent‐space diagnostics, we computed 3D PCA on the generated latents (aggregated per mapping and also per case) and exported the projections. The resulting clouds confirm distributional equivalence between device and PRNG‐iid latents and clear separation from degraded controls (Figure S44). Finally, to verify independence between the spatial PUF mean anchor and temporal TRNG noise, we compared co‐localized and de‐localized pairing protocols under matched preprocessing. Generative metrics are unchanged across protocols, and raw device statistics show weak coupling (Figures S39 and S40). This two‐dimensional MNIST workflow was used only as a conceptual demonstration of latent‐space controllability and was separate from the 512‐dimensional StyleGAN2‐AFHQv2 animal‐image inference.

### 4.11 Hardware‐Derived Latent‐Vector Construction and StyleGAN2‐AFHQv2 Inference

Transient‐current traces measured from nine mixed‐ZGO devices were used as candidate hardware sources. For each inference, two device traces, *a*and *b*, were selected, and a common starting index *t*_0_was defined. A total of 256 consecutive current samples were extracted from each selected trace. Each measured sample was linearly normalized from the current range of 5–30 µA to the latent‐coordinate range of −2 to 2 according to:

(15) $$\mathrm{Normalize}\left(I\right)=4\frac{I-5\;\mu A}{30\;\mu A-5\;\mu A}-2$$

The normalized samples were assigned to the latent coordinates according to:

(16) $$z\;\left[2k\right]=\mathrm{Normalize}\left(I_{a}\left[t_{0}+k\right]\right)\;$$

(17) $$z\left[2k+1\right]=\mathrm{Normalize}\left(I_{b}\left[t_{0}+k\right]\right)$$

where *k* = 0, 1, …, 255. Thus, each 512‐dimensional latent vector contained 256 measured samples from each of two selected device traces. No scalar latent value was repeated to fill the vector, and no software‐generated random value was used to populate the latent coordinates. The even/odd assignment was a deterministic coordinate‐placement rule. The latent vector was reshaped to [1, 512] and supplied to *G*_ema_ of the pretrained *stylegan2‐afhqv2‐512×512.pkl* model. Inference was performed using *class_label* = *None*, *truncation_psi* = 0.7, and *noise_mode* = *const*, with gradient computation disabled. The pretrained generator was used without retraining or fine‐tuning. Therefore, image synthesis was performed by the pretrained StyleGAN2‐AFHQv2 model, while the mixed‐ZGO measurements provided the hardware‐derived latent input.

### 4.12 Data Sources

The NIST analysis used 20 device‐derived bitstreams containing 60 000 bits per device and did not use an external image dataset. The image‐generation validation used the public MNIST and AFHQv2 datasets together with the pretrained 512 × 512 StyleGAN2‐AFHQv2 generator. To document the image‐generation workflow in a concise and reproducible form, the TRNG digitization, NIST evaluation, hardware‐derived latent‐vector construction, and StyleGAN2‐AFHQv2 inference procedures are described in the “Reproducibility Pseudocode” section (Supporting Information).

## Author Contributions

**Seungme Kang**: writing – original draft, conceptualization, methodology, investigation, visualization. **Wangmyung Choi**: writing – original draft, methodology, conceptualization, investigation, visualization. **Jueun Lee**: software, methodology, investigation, writing – original draft. **Yeong Kwon Kim**: methodology, investigation, formal analysis, software. **Byung Chul Jang**: software, project administration, writing – review and editing, supervision. **Wonjun Shin**: funding acquisition, software, writing – review and editing, project administration, supervision. **Hocheon Yoo**: funding acquisition, writing – review and editing, conceptualization, project administration, supervision.

## Conflicts of Interest

The authors declare no conflicts of interest.

## Supporting information




**Supporting File**: advs78054‐sup‐0001‐SuppMat.docx.

## Data Availability

The data that support the findings of this study are available from the corresponding author upon reasonable request.
