# Supplementary material for: Dual Randomness From Spatial and Temporal Variations in Stochastic Zinc Gallate Nanoclusters for PUF and TRNG Co‐Driven Image Generation
Source: Adv Sci (Weinh). 2026 Sep 28:e78054. Online ahead of print. doi: 10.1002/advs.78054 (PMC13620269; doi:10.1002/advs.78054)
Supplement: Supplementary file 1 [file ADVS-9999-e78054-s001.docx]

Supporting Information

**Dual Randomness from Spatial and Temporal Variations in Stochastic Zinc Gallate Nanoclusters for PUF and TRNG Co-Driven Image Generation**

Seungme Kang^1,†^, Wangmyung Choi^1,†^, Jueun Lee^2,†^, Yeong Kwon Kim^3^,

Byung Chul Jang^3,*^, Wonjun Shin^2,4,*^ and Hocheon Yoo^1,5,6,*^

^†^These authors contributed equally to this work.

S. Kang, W. Choi, Prof. H. Yoo
^1^Department of Electronic Engineering, Hanyang University, Seoul 04763, Republic of Korea

^*^E-mail: hocheon@hanyang.ac.kr

J. Lee, Prof. W. Shin

^2^Department of Electrical and Computer Engineering, Sungkyunkwan University (SKKU), Suwon 16419, Republic of Korea

^*^E-mail: swj0107@skku.edu

Y. K. Kim, Prof. B.C. Jang
^3^School of Electronic and Electrical Engineering, Kyungpook National University, Daegu 41566, Republic of Korea

^*^E-mail: bc.jang@knu.ac.kr

Prof. W. Shin
^4^Department of Semiconductor Convergence Engineering, Sungkyunkwan University (SKKU), Suwon 16419, Republic of Korea

^*^E-mail: swj0107@skku.edu

Prof. H. Yoo
^5^Department of Artificial Intelligence Semiconductor Engineering, Hanyang University, Seoul 04763, Republic of Korea

^*^E-mail: hocheon@hanyang.ac.kr

Prof. H. Yoo
^6^Hanyang Institute for Quantum Science and Quantum Technology, Hanyang University, Seoul 04763, Republic of Korea

^*^E-mail: hocheon@hanyang.ac.kr

**<Table of Contents>**

**Supporting Figures**

Figure S1. EDS mapping of a mixed ZGO NC-based device.	S5

Figure S2. Physical characteristics of the materials comprising the mixed-ZGO NCs-based device.	S6

Figure S3. Schematic diagram of energy band diagram.	S7

Figure S4. Mechanism of the device according to band structure type.	S8

Figure S5. XRD spectra of different ZGO NCs sizes.	S9

Figure S6. Schematic illustration of interaction between ZGO nanoparticles and $\mathrm{NH}_{4}^{+}$ ions.	S10

Figure S7. Measurement conditions of pH, Zeta potentials, and DLS size distribution during ZGO NCs synthesis.	S11

Figure S8. AFM mapping of various conditions with different ZGO NCs sizes.	S12

Figure S9. Current-voltage characteristics with noise measurements reflected according to ZGO NCs sizes.	S13

Figure S10. LFN characteristics of devices at different locations within a 10 × 20 array.	S14

Figure S11. Security performance parameters of the single TRNG device.	S15

Figure S12. Capacitance-voltage characteristics of devices at different locations.	S16

Figure S13. Current-voltage characteristics of devices at different locations.	S17

Figure S14. Impedance characteristics of devices at different locations.	S18

Figure S15. UPS spectra of the secondary cut-off region of devices at different locations.	S19

Figure S16. PUF bit maps obtained from 10 consecutive read measurements.	S20

Figure S17. Distribution of the intra-HD obtained from 10 repeated measurements of the 10 × 10 PUF array.	S21

Figure S18. Inter-device uniqueness of 10 independently fabricated PUF devices.	S22

Figure S19. Spatial independence of the PUF responses.	S23

Figure S20. Initial current-voltage characteristics of 200 devices.	S24

Figure S21. Current-voltage characteristics of 200 devices after 91 days.	S25

Figure S22. Comparison for evaluating the long-term stability of PUF.	S26

Figure S23. Thermal stability of the PUF output.	S27

Figure S24. Humidity stability of the PUF output.	S28

Figure S25. Current-voltage characteristics repeatedly measured from the same PUF cell/device over 1,000 cycles	S29

Figure S26. PUF cell operational stability.	S30

Figure S27. Bias-stress stability of a PUF cell.	S31

Figure S28. Current noise characteristics of TRNG after 237 days.	S32

Figure S29. Comparison for evaluating the long-term stability of TRNG security code.	S33

Figure S30. Environmental dependence of the low-frequency-noise characteristics and TRNG outputs of a representative mixed-ZGO NC device.	S34

Figure S31. Test-set occupancy across eight calibrated quantile intervals.	S35

Figure S32. NIST statistical evaluation of 20 quantile-encoded device-derived bitstreams.	S36

Figure S33. Two-dimensional latent map of the MNIST GAN trained for each numeric class.	S38

Figure S34. Correlation between the noise levels generated from a single TRNG and the generation of MNIST images.	S39

Figure S35. Mapping of measured transient-current samples and construction of the 512-dimensional hardware-derived latent input.	S40

Figure S36. Comparison of the generative quality between hardware TRNG (device) and software PRNG (ideal-i.i.d. Gaussian) under different normalization mappings.	S42

Figure S37. Calculated parameters of the generative quality according to simulated software PRNG type (LCG4 and XOR4).	S44

Figure S38. Relationship between device selection based on normalized output and the frequency of mode images.	S45

Figure S39. Generative quality metrics of co-localized and de-localized pairing.	S47

Figure S40. Scatter plots of raw device TRNG statistics (per channel).	S48

Figure S41. Truncation-*ψ* sweep results for FID of PRNG and TRNG across *ψ* = 0.4–1.0.	S49

Figure S42. Truncation-*ψ* sweep results for KID of PRNG and TRNG across *ψ* = 0.4–1.0.	S50

Figure S43. Truncation-*ψ* sweep results for Inception-feature-space diversity proxy of PRNG and TRNG across *ψ* = 0.4–1.0.	S51

Figure S44. Latent PCA analysis of generated vectors.	S52

Figure S45. Comparison of environment stability test for PUFs and TRNGs.	S53

**Supporting Table**

Table S1. Security parameters of the TRNG outputs under different environmental conditions.	S54

**References**

**Reproducibility Pseudocode**

**Figure S1.** EDS mapping image of a mixed ZGO NC-based device (Ti: yellow, S: purple, O: orange, Zn: green, Ga: cyan)

**Figure S2.** Physical characteristics of the materials comprising the mixed-ZGO NCs-based device: (a) UV-vis spectra of DNTT and mixed-ZGO NCs, Tauc plot extracted from the UV-vis spectrum of (b) DNTT and (c) mixed-ZGO, and (d) UPS spectrum of DNTT.

**Figure S3.** Schematic diagram of energy band diagram for n-Si, mixed-ZGO NCs, DNTT, and Ti.

**Figure S4.** Mechanism of the device based on band structure: band diagram of the DNTT/n-Si structure under (a) reverse bias and (b) forward bias, band diagram of the DNTT/mixed-ZGO NCs/n-Si structure under (c) reverse bias and (d) forward bias.

**Figure S5.** Normalized XRD spectra of (a) small and (b) large ZGO NCs synthesized with different reaction times.

**Figure S6.** Schematic illustration of interaction between ZGO nanoparticles and $\mathrm{NH}_{4}^{+}$ ions: (a) small ZGO NCs synthesized for 24 h and (b) large ZGO NCs synthesized for 36 h, showing that extended reaction times increase the concentration of $\mathrm{NH}_{4}^{+}$ ions, resulting in the formation of larger ZGO NCs.

**Figure S7.** Photographs of pH measurement conditions during the synthesis of ZGO NCs at (a) 24 h and (b) 36 h. (c) Zeta potentials of small and large ZGO NC solutions redispersed in a chloroform/ethanol (3:1 v/v) mixture solvent. (d) DLS size distributions of small and large ZGO NCs.

**Figure S8.** AFM mapping images for different conditions with (a) small-ZGO NCs, (b) large-ZGO NCs, and (c) mixed-ZGO NCs.

**Figure S9.** Current-voltage characteristics with noise measurements reflected according to the composition conditions of (a) small ZGO NCs and (b) large ZGO NCs for 10 devices.

**Figure S10.** Measured current power spectral density of 15 devices at different locations within a 10×20 array composed of mixed ZGO NCs.

**Figure S11.** Security performance parameters obtained from the illustrative binary TRNG codes based on mixed-ZGO NCs: (a) uniformity (49.89%), (b) inter-HD (51.33%), (c) entropy (average: 0.91), and (d) bit-aliasing (49.45%).

**Figure S12.** Capacitance-voltage characteristics measured at 4 different positions of (a) large- and (b) small-ZGO NCs devices.

**Figure S13.** Current-voltage characteristics measured at 4 different positions of (a) large-, (b) small-, and (c) mixed-ZGO NCs devices under alternating current conditions.

**Figure S14.** Impedance characteristics measured at 4 different positions of (a) large- and (b) small-ZGO NCs devices.

**Figure S15.** UPS spectra of the secondary cut-off region measured at four arbitrarily selected device positions.

**Figure S16.** PUF bit maps obtained from 10 consecutive read measurements of the 10 × 10 array.

**Figure S17.** Distribution of the intra-HD obtained from 10 repeated measurements of the 10 × 10 PUF array. The mean and standard deviation of the intra-HD were 0.20% and 0.42%, respectively.

**Figure S18.** Inter-device uniqueness of 10 independently fabricated PUF devices. (a) Pairwise inter-device HD heatmap obtained from 10 independently fabricated PUF devices. (b) Distribution of the 45 inter-device HD values, showing a mean of 50.40% and a standard deviation of 4.40%.

**Figure S19.** Spatial correlation analysis of the PUF responses. (a) Spatial distribution of the current magnitude measured at −1 V across the PUF array. (b) Two-dimensional lagged Pearson correlation coefficient heatmap as a function of row and column lags. (c) Lagged Pearson correlation coefficients along the horizontal and vertical directions as a function of spatial lag.

**Figure S20.** Current–voltage characteristics of a 10 × 20 array device based on mixed-ZGO NCs from position 1 to 200 at the pristine state.

**Figure S21.** Current–voltage characteristics of a 10 × 20 array device based on mixed-ZGO NCs from position 1 to 200 after 91 days.

**Figure S22.** Comparison for evaluating the long-term stability of PUF security code. Bit-mapping of the security code in its (a) initial state and (b) after 91 days. (c) Inter-cell HD (46.21%) and (d) entropy (avg: 0.83) after 91 days. All PUF metrics exhibit 0% bit and stay at their initial values, demonstrating long-term stability.

**Figure S23.** Thermal stability of the PUF output. (a) PUF key mappings obtained from the 4 × 4 device array under different temperature conditions of RT, 40 °C, 60 °C and 80 °C. (b) I-V characteristics measured under four temperature conditions.

**Figure S24.** Humidity stability of the PUF output. (a) PUF key mappings obtained from the 4 × 4 device array under different RH conditions of approximately 60%, 81%, and 90% RH. (b) I-V characteristics measured under three humidity conditions.

**Figure S25.** Current-voltage characteristics repeatedly measured from the same PUF cell/device over 1,000 cycles; (a) 1–250, (b) 251–500, (c) 501–750, and (d) 751–1,000 cycles.

**Figure S26.** (a) Current at −1 V obtained from 1,000 repeated current-voltage measurements of the same PUF cell/device. (b) Distribution of the extracted current values over 1,000 cycles at −1 V.

**Figure S27.** (a) Current-time characteristics of the same PUF cell/device under a constant bias of – 1 V for 1,000 s. (b) Current-voltage characteristics measured at the initial condition and after the 1,000 s bias stress.

**Figure S28.** Comparison for evaluating the long-term stability of random number generation. Time-domain TRNG stability after 237 days.

**Figure S29.** Comparison for evaluating the long-term stability of TRNG security code. Bit-mapping of the security code (a) after 237 days. (b) Uniformity (51.11%), (c) inter-HD (50.20%) and (d) entropy (avg: 0.90) after 237 days. All TRNG metrics remain within less than 3% variation and close to the ideal values, confirming long-term stability.

**Figure S30.** Environmental dependence of the low-frequency-noise characteristics and TRNG outputs of a representative mixed-ZGO NC device. Normalized current-noise power spectral densities, *S*_I_/*I*^2^, measured at −1 V, −0.5 V, 0.5 V, and 1 V under (a) ambient condition, (b) a vacuum condition of approximately 20 Torr at RT, and (c) high temperature of 60 °C. 30 × 30 TRNG bit maps extracted at −1 V for each environment using a quantile-based encoding scheme that accounts for hardware non-idealities: (d) ambient condition, (e) approximately 20 Torr at RT, and (f) high temperature at 60 °C.

**Figure S31.** Test-set occupancy of the eight quantile-defined output intervals obtained using frozen calibration parameters. For each transient-current trace, the first 2,222 of 22,222 chronological samples were used exclusively to determine the calibration median and seven quantile boundaries at cumulative probabilities of 1/8, 2/8, ..., and 7/8. These parameters were frozen and applied without modification to the remaining 20,000 test samples. No median or quantile boundary was recalculated using the test segment. The heatmap shows the percentage of test samples assigned to each bin under ambient, vacuum, and 60 °C conditions at −1 V. The ideal occupancy is 12.5% per bin.

**Figure S32.** Statistical evaluation of the 20 device-derived 60,000-bit sequences using the 11 applicable NIST criteria. Random Excursions and Random Excursions Variant were excluded from the pass assessment because the available sequences did not satisfy the recommended input-length requirements. No temporal decimation, digital filtering, or post-processing was applied, and *p* = 0.01 was used as the acceptance threshold. (a) Summary of the 11 reported NIST criteria, (b) monobit test, (c) frequency within a block test, (d) runs test, (e) longest run of ones in a block test, (f) binary matrix rank test, (g) discrete Fourier transform test, (h) non-overlapping template matching test, (i) serial test, (j) approximate entropy test, (k) cumulative sums test—forward, (l) cumulative sums test—backward.

**Supporting Discussion: Additional details of the TRNG bit-generation procedure.**

For each measured trace, the median and seven quantile boundaries were calculated before bit encoding and used for the subsequent bit encoding; the thresholds were not adjusted or optimized according to the NIST results. Each fluctuation sample was assigned to one of eight intervals, converted into a 3-bit natural-binary word from 000 to 111, and concatenated in chronological order. No temporal decimation (*D* = 1) or additional post-processing, including Von Neumann correction, XOR whitening or mixing, digital filtering, or error-correction coding, was applied. The single-threshold binary map shown in Figure 3b was used only for visual illustration and was not used to generate the NIST input. The NIST evaluation was conducted using 20 device-derived bitstreams containing 60,000 bits each, with an acceptance threshold of *p* = 0.01. Each sequence corresponded to 20,000 measured current samples and 6.25 s of retained measurement data.

**Figure S33.** (a) Two-dimensional latent map of the trained MNIST GAN showing natural clustering of each digit class. (b) Individual latent maps for digits 0 (b-1) through 9 (b-10), respectively, further illustrating the characteristic clustering in the latent space.

**Supporting Discussion: Latent map of the trained MNIST GAN.**

Figure S33 shows the latent map of the trained MNIST GAN. Figure S33a presents a two-dimensional plot (*Z*_1_ vs. *Z*_2_) where different colors represent distinct digit categories, demonstrating that each digit forms a characteristic cluster in the latent space. Figures S33(b-1) to (b-10) show individual latent maps corresponding to digits 0 through 9, respectively, further confirming that the inherent clustering in the latent space can be exploited if a controlled sampling method is applied.

**Figure S34.** (a) Latent space distributions of generated MNIST images for sigma values of 0.03 (a-1), 0.1 (a-2), 0.3 (a-3), and 1.0 (a-4), respectively, demonstrating the effect of increasing noise on vector spread. (b) Class distribution of 1,000 generated MNIST images (using a CNN-based classifier) corresponding to the sigma values in (a); low sigma results in homogeneous outputs, whereas high sigma produces a uniform distribution across all classes.

**Supporting Discussion: Effect of varying the noise parameter on latent space sampling in a simple GAN trained on MNIST.**

Figure S34 shows the effects of varying the noise parameter (σ) on latent space sampling in a simple GAN trained on MNIST. Figure S34a presents the two-dimensional latent space projections obtained when latent vectors are sampled using four different sigma values: 0.03 (a-1), 0.1 (a-2), 0.3 (a-3), and 1.0 (a-4), respectively. Figure S34b present the classification results of 1,000 generated images using a CNN-based classifier with over 99.5% accuracy. Figure S34(b-1) shows that with σ = 0.03, almost all generated images are classified into a single digit (for example, digit “7”), demonstrating that very low noise yields homogeneous outputs. Figures S34(b-2) to (b-4) present the classification distributions for increasing σ values. These results indicate that while higher σ values enable a wider range of image classes to be generated, they also fail to selectively favor any particular class—thus underlining the need for a method that combines high entropy with deterministic mean control.

**Figure S35.** (a) Transient-current traces measured from nine candidate mixed-ZGO devices at a sampling frequency of 3,200 samples s^−1^, with mean values ranging from 5 to 30 μA. (b) Linear mapping of the measured current samples to the latent-coordinate range from −2 to 2. For each 512-dimensional StyleGAN2-AFHQv2 latent vector, two traces were selected, and 256 consecutive normalized samples from each trace were assigned to the even- and odd-indexed coordinates, respectively.

**Supporting Discussion: Mapping of hardware-measured transient current responses onto the latent space.**

Figure S35 shows the mapping of experimentally measured transient-current samples and the construction of the hardware-derived latent input. Figure S35a presents nine candidate device traces recorded at a sampling frequency of 3,200 samples s^−1^. To construct one 512-dimensional latent vector, two traces, $a$ and $b$, were selected, and 256 consecutive current samples were extracted from each trace from a common starting index *t*_0_. Figure S35b shows how each measured sample was linearly normalized from the experimentally observed current range of 5–30 μA to the latent-coordinate range of −2 to 2 using Equation (S1):

$Z_{1} , Z_{2}=\frac{I-17.5 \mu A}{6.25 \mu A}$	(S1)

scaling them to a range between –2 and 2. This normalization is easily implemented via a simple op-amp circuit (voltage subtractor and divider), which enables the hardware measurements to be accurately reflected in the latent space. The same normalization procedure is applied in our simulation process to ensure consistency between experimental data and simulated latent vector sampling. The normalized samples were assigned to the latent vector according to:

$z[2k]=\mathrm{Normalize}\left( I_{a}[t_{0}+k] \right)$	(S2)$z[2k+1]=\mathrm{Normalize}\left( I_{b}[t_{0}+k] \right)$	(S3)

where *k* = 0, 1, …, 255. Accordingly, all 512 latent coordinates originate from 512 measured temporal-current sample instances: 256 samples from device $a$ and 256 samples from device $b$. In terms of physical hardware sources, each vector contains measurements from only two device channels selected from the nine candidate traces. No coordinate is generated by repeating a scalar *Z*_1_ or *Z*_2_ value, and no coordinate is populated using a software-generated random number. The deterministic operations are the fixed linear normalization and even/odd coordinate assignment. The resulting latent vector was supplied to the pretrained StyleGAN2-AFHQv2 model (*stylegan2-afhqv2-512x512.pkl*) using *truncation_psi* = 0.7 and *noise_mode* = *const*. The generator was used only for inference, without retraining or fine-tuning. Thus, the pretrained generator performed image synthesis, whereas the mixed-ZGO measurements supplied the hardware-derived latent input.

**Figure S36.** Comparison of the generative quality of images synthesized using hardware-derived latent inputs and software-PRNG latent inputs (ideal i.i.d. Gaussian) under different normalization mappings. FID of (a) TRNG and (b) PRNG. KID of (c) TRNG and (d) PRNG. The Inception-feature-space diversity proxy is shown in (e) for the hardware-derived latent inputs and in (f) for the software-PRNG latent inputs. Linear removes the mean and rescales the variance with bounded clipping (hardware-friendly); Rank applies a normal-scores transform (empirical CDF to standard normal); and Qmatch aligns heavy-tailed sources to a target distribution via z-scoring with mild saturation. These three preprocessing routes were evaluated as a separate sensitivity benchmark and should not be interpreted as the fixed min–max mapping used for the principal Figure 4 latent-vector construction.

**Figure S37.** Calculated parameters of the generative quality according to simulated software PRNG type: LCG4 and XOR4 under different normalization mappings. FID of (a) LCG4 and (b) XOR4. KID of (c) LCG4 and (d) XOR4. The Inception-feature-space diversity proxy is shown in (e) for LCG4 and in (f) for XOR4.

**Figure S38.** Separate two-dimensional MNIST latent-space proof-of-concept. (a) Device-pair selection for two-dimensional latent sampling, where the horizontal and vertical axes denote the devices associated with the *Z*_1_ and *Z*_2_ coordinates, respectively, and the predominant generated digit is indicated for each pair. (b) Frequency distribution of the predominant digit across device pairs, with a frequency of 100 indicating complete reproducibility under the evaluated conditions. (c) Example MNIST digits generated from selected regions of the two-dimensional latent space. The *Z*_1_ and *Z*_2_ coordinates in this analysis are separate from the 512-dimensional StyleGAN2-AFHQv2 latent-vector construction.

**Supporting Discussion: Relationship between device selection and image generation.**

Figure S38 shows the relationship between device-pair selection and the generated digit class in the separate two-dimensional MNIST proof-of-concept. Figure S38a presents a plot in which the horizontal axis corresponds to the device number selected for *Z*_1_ and the vertical axis to the device number selected for *Z*_2_. The plot indicates the mode (most frequent) image generated for each device pair, demonstrating that the selected combination of devices directly determines the predominant image category. Figure S38b shows the frequency distribution of the mode images, where a frequency of 100 indicates that the same image appears in all generated samples. Deviations from this value are observed at the boundaries of the latent space due to variations in device output. Figure S38c presents example images generated for target categories (digits 0–9) based on a specific region (highlighted by a red square in Figure S38a). These images confirm that by selecting the appropriate region in the latent space through proper device pairing, it is possible to reproducibly generate images from a specific category with subtle variations in features. The *Z*_1_ and *Z*_2_ axes in Figure S38 represent the two coordinates of the MNIST latent space and should not be interpreted as two scalar values repeated to populate the 512-dimensional StyleGAN2-AFHQv2 input. For the animal-image inference, the 512-dimensional vector instead contains 256 consecutive measured samples from each of two selected device traces, as described in Figure S35.

**Figure S39.** Comparison of image-evaluation metrics between co-localized and de-localized pairing protocols. In the co-localized condition, the mean-current and temporal-fluctuation components were obtained from the same device trace. In the de-localized condition, the mean-current component from one device was combined with the temporal-fluctuation component from a different device, with matched device usage, sample counts, preprocessing, and generator conditions. (a) FID, (b) KID, and (c) the Inception-feature-space diversity proxy.

**Figure S40.** Scatter plots of measured TRNG statistics for each channel. (a) Mean current, 𝑢_𝐼_, versus lag-1 autocorrelation, ACF1. (b) Mean current, 𝑢_𝐼_, versus normalized standard deviation, 𝜎_𝐼_/𝑢_𝐼_. The weak correlations indicate weak coupling under the evaluated conditions.

**Figure S41.** Truncation-*ψ* sweep of FID for images synthesized using software-PRNG and hardware-derived latent inputs across *ψ* = 0.4–1.0.

**Figure S42.** Truncation-*ψ* sweep of KID for images synthesized using software-PRNG and hardware-derived latent inputs across *ψ* = 0.4–1.0.

**Figure S43.** Truncation-ψ sweep results for the Inception-feature-space diversity proxy of PRNG and TRNG across ψ = 0.4–1.0.

**Figure S44.** PCA analysis of latent vectors constructed from (a) hardware-derived current inputs and (b) software i.i.d.-Gaussian PRNG inputs.

**Figure S45.** Comparison of stability tests for (a) PUFs and (b) TRNGs under different environmental conditions.

**Table S1.** Uniformity, inter-HD, and entropy of the TRNG outputs under different environmental conditions.

| **Environment** | **Uniformity [%]** | **inter-HD [%]** | **Entropy** |
| --- | --- | --- | --- |
| Ambient condition (RT, 760 Torr) | 51.05 | 50.06 | 0.91 |
| Vacuum (RT, 20 Torr) | 49.51 | 49.82 | 0.94 |
| Temperature (60 °C, 760 Torr) | 49.44 | 50.17 | 0.96 |

**Table S2.** Comparative analysis of key parameters in PUF and TRNG security device papers.

| **Device type** | **Security type** | **Uniformity [%]** | **Inter-HD [%]** | **Intra-HD [%]** | **Entropy** | **Long-term stability [day]** | **Environmental stability test** | **NIST** | **Applications** | **[Ref]** |
| --- | --- | --- | --- | --- | --- | --- | --- | --- | --- | --- |
| OLED | PUF | 50.3 | 49.01 | ≈ 0 | N/A | 9 months | Mechanical abrasion and washing test | N/A | N/A | [S1] |
| MTJ | PUF | N/A | 50.2 | N/A | 0.98 | N/A | Thermal: –40 – 150 °C | PASS | GAN | [S2] |
| Transistor | PUF | 49 | ≈ 49.4 | N/A | 0.99 | 5 | N/A | N/A | N/A | [S3] |
| RRAM array | PUF | 50 | 50.94 | N/A (BER=0%) | N/A | N/A | N/A | N/A | Encryption-decryption | [S4] |
| FTJ | PUF | N/A | 48.83 | 4.17 | N/A | N/A | N/A | PASS | N/A | [S5] |
| APT | PUF | 49.59 | 49.65 | 3.23 | N/A | N/A | N/A | PASS | Wearable device | [S6] |
| PD | TRNG | 53.90 | 50.37 | N/A | 0.98 | 530 | Thermal: 40 – 100 °C Humidity: 30 – 80% RH | PASS | GAN | [S7] |
| Memristor | TRNG | ≈ 50 | N/A | N/A | N/A | N/A | N/A | PASS | Encryption-decryption | [S8] |
| Transistor | TRNG | N/A | N/A | N/A | N/A | N/A | N/A | PASS | N/A | [S9] |
| Memristor | TRNG | N/A | N/A | N/A | N/A | 12,000 s | Thermal: 285 – 355 K | PASS | N/A | [S10] |
| Transistor | TRNG | N/A | N/A | N/A | N/A | N/A | Thermal: 300 – 360 K | PASS | N/A | [S11] |
| Gated silicon diode | TRNG | 51 | 49.98 | 49.79 | N/A | N/A | N/A | PASS | N/A | [S12] |
| SRAM array | PUF $+$ TRNG | N/A | 50.3 (PUF) | 1.6, 3.4 (PUF) | 0.99 (PUF) 0.99 (TRNG) | N/A | Thermal: –25 – 100 °C (PUF) Thermal: –25 – 100 °C (TRNG) | PASS (TRNG) | N/A | [S13] |
| Flash memory circuit | PUF $+$ TRNG | 50.35 (PUF) | 49.99 (PUF) | N/A | 0.99 (PUF) 0.99 (TRNG) | N/A | Thermal: 25 – 85 °C (PUF) | PASS (PUF) PASS (TRNG) | Machine learning modeling attacks | [S14] |
| Photo transistor | PUF $+$ TRNG | 50 (PUF) 50.01 (TRNG) | 50.27 (PUF) | 1.42 (PUF) | 1 (PUF) 0.99 (TRNG) | 365 (PUF) 20 months (TRNG) | Thermal: 25, 70 °C Humidity: 30, 70% RH (PUF) Thermal: 25, 70 °C Humidity: 30, 70% RH (TRNG) | PASS (TRNG) | N/A | [S15] |
| Vertically Integrated Transistor | PUF $+$ TRNG | 50 (PUF) | 50.67 (PUF) | N/A | N/A | 2 months (PUF) | N/A | PASS (TRNG) | N/A | [S16] |
| Memristor array | PUF $+$ TRNG | N/A | 50 (PUF) | 0 (PUF) | N/A | N/A | N/A | PASS (PUF) PASS (TRNG) | Encryption-decryption | [S17] |
| Memristor array | PUF $+$ TRNG | 50.09 (PUF) 49.99 (TRNG) | 50.06 (PUF) | 0 (PUF) | 0.99 (TRNG) | N/A | Thermal: –40 – 125 °C (PUF) Thermal: –40 – 125 °C (TRNG) | PASS (PUF) PASS (TRNG) | Identity authentication | [S18] |
| Irregular material junction diode | PUF $+$ TRNG | 49.89 (TRNG) | Cell; 46.21 Device; 50.40 (PUF) 51.33 (TRNG) | 0.20 (PUF) | 0.83 (PUF) 0.91 (TRNG) | 91 (PUF) 237 (TRNG) | Thermal: 25 – 80 °C Humidity: 60 – 90% RH (PUF) Thermal: 25, 60 °C Pressure: 20, 760 Torr (TRNG) | PASS (TRNG) | GAN | This work |

* OLED: organic light-emitting diode, MTJ: magnetic tunnel junction, GAN: generative adversarial network, RRAM: resistive random-access memory, BER: bit error rate, FTJ: ferroelectric tunnel junction, APT: Ag-paste tag, PD: photodetector, SRAM: static random access memory.

**References**

[S1] N. Kayaci, R. Ozdemir, M. Kalay, N. B. Kiremitler, H. Usta, M. S. Onses, Advanced Functional Materials 2022, 32, 2108675.

[S2] J. Kang, D. Han, K. Lee, S. Ko, D. Koh, C. Park, J. Ahn, M. Yu, M. Pakala, S. Noh, ACS nano 2024, 18, 12853.

[S3] S. Ghosh, Y. Zheng, S. S. Radhakrishnan, T. F. Schranghamer, S. Das, Nano letters 2023, 23, 5171.

[S4] J. M. Cho, S. S. Kim, T. W. Park, D. H. Shin, Y. R. Kim, H. J. Park, D. Y. Kim, S. H. Lee, T. Park, C. S. Hwang, Nanoscale horizons 2025, 10, 113.

[S5] J. Yu, K. K. Min, Y. Kim, S. Kim, S. Hwang, T.-H. Kim, C. Kim, H. Kim, J.-H. Lee, D. Kwon, Nanotechnology 2021, 32, 485202.

[S6] M. S. Kim, M. H. Kang, J. S. Kim, Y. K. Hong, G. J. Lee, IEEE Journal of Selected Topics in Quantum Electronics 2023, 30, 1.

[S7] T. Park, J. Seo, N. Kim, C. Kim, Y. J. Kim, H. Kim, H. H. Kim, S. Oh, D. C. Kim, D. Son, Advanced Materials 2025, 37, 2419579.

[S8] W. H. Cheong, J. H. In, J. B. Jeon, G. Kim, K. M. Kim, Nature Communications 2024, 15, 6318.

[S9] S. Kim, M.-S. Kim, Y. Lee, H.-D. Kim, Y.-K. Choi, S.-J. Choi, IEEE Access 2021, 9, 91341.

[S10] K. S. Woo, A. Zhang, A. Arabelo, T. D. Brown, M. Park, A. A. Talin, E. J. Fuller, R. S. Bisht, X. Qian, R. Arroyave, Nature communications 2024, 15, 4656.

[S11] S.-I. Kim, H.-J. You, M.-S. Kim, U.-S. An, M.-S. Kim, D. H. Lee, S.-T. Ryu, Y.-K. Choi, Science Advances 2024, 10, eadk6042.

[S12] J. Jeon, Y. Shin, H. Heo, J. Son, S. Ryu, K. Cho, S. Kim, Science China Information Sciences 2025, 68, 162401.

[S13] S. Taneja, V. K. Rajanna, M. Alioto, IEEE Journal of Solid-State Circuits 2021, 57, 153.

[S14] S. Larimian, M. R. Mahmoodi, D. B. Strukov, IEEE Transactions on Electron Devices 2020, 67, 1586.

[S15] R. Ji, W. Huang, B. Liang, X. Wu, H. Hu, J. Hu, J. Liu, H. Wu, S. Feng, [W. Huang](https://scholar.google.co.kr/citations?user=s8gQQKsAAAAJ&hl=ko&oi=sra), Advanced Materials 2026, 38(7), e09239.

[S16] J.W. Lee, [J.K. Han](https://scholar.google.co.kr/citations?user=q80z91YAAAAJ&hl=ko&oi=sra), [S.I. Kim](https://scholar.google.co.kr/citations?user=LlzCK_sAAAAJ&hl=ko&oi=sra), H.Y. Maeng, [S.Y. Yun](https://scholar.google.co.kr/citations?user=gtxatC8AAAAJ&hl=ko&oi=sra), [J.H. Son](https://scholar.google.co.kr/citations?user=6zFtr2oAAAAJ&hl=ko&oi=sra), [S.W. Lee](https://scholar.google.co.kr/citations?user=_5RFbe4AAAAJ&hl=ko&oi=sra), [Y.K. Choi](https://scholar.google.co.kr/citations?user=L3xBe_4AAAAJ&hl=ko&oi=sra), Nano Research 2025, 18(1), 94907045.

[S17] [X. Li](https://scholar.google.co.kr/citations?user=jGB4yqcAAAAJ&hl=ko&oi=sra), [B. Gao](https://scholar.google.co.kr/citations?user=czP0vJoAAAAJ&hl=ko&oi=sra), [Q. Qin](https://scholar.google.co.kr/citations?user=krv0LagAAAAJ&hl=ko&oi=sra), [P. Yao](https://scholar.google.co.kr/citations?user=wydAfisAAAAJ&hl=ko&oi=sra), J. Li, [H. Zhao](https://scholar.google.co.kr/citations?user=KXBJ_BUAAAAJ&hl=ko&oi=sra), C. Liu, [Q. Zhang](https://scholar.google.co.kr/citations?user=7aAIE6MAAAAJ&hl=ko&oi=sra), Z. Hao, Y. Li, D. Kong, J. Xu, J. Yang, J. Tang, Y. Niu, X. Yan, H. Qian, H. Wu, Nature Electronics 2025, 8(6), 518-528.

[S18] X. Li, B. Lin, B. Gao, Y. Lu, S. Yang, Z. Su, T.-Y. Shen, J. Tang, H. Qian, H. Wu, Science Advances 2025, 11, eadr0112.

**Reproducibility Pseudocode**

Data and Code Availability Response Package

=================================================

Dataset clarification

---------------------

NIST input: 20 author-generated device bitstreams, 60,000 bits per device. No image dataset is used for NIST testing.

MNIST: loaded through torchvision.datasets.MNIST for the two-dimensional latent-space proof of concept.

AFHQv2: public 512 x 512 cat, dog, and wildlife dataset underlying the pretrained StyleGAN2-AFHQv2 animal generator.

Pretrained model: stylegan2-afhqv2-512x512.pkl.

Algorithm S1. Conversion of measured transient current into a TRNG bitstream

----------------------------------------------------------------------------

INPUT: Measured transient-current samples I[0 ... N-1]

PARAMETERS: Sampling frequency fs = 3,200 samples/s; decimation factor D = 1

OUTPUT: Binary sequence B

m <- MEDIAN(I)

FOR n <- 0 TO N-1:

d[n] <- ABS(I[n] - m)

q[1 ... 7] <- EMPIRICAL_QUANTILES(d, probabilities = {1/8, 2/8, ..., 7/8})

B <- empty sequence

FOR n <- 0 TO N-1:

label <- QUANTILE_INTERVAL_INDEX(d[n], q)	# integer from 0 to 7

word <- INTEGER_TO_BINARY(label, width = 3)	# 000 through 111

APPEND B WITH word

RETURN B

Effective bit-generation rate = 3,200 samples/s x 3 bits/sample = 9,600 bit/s.

No Von Neumann correction, XOR whitening or mixing, digital filtering, or error-correction coding is applied.

Algorithm S2. NIST statistical evaluation of device-derived bitstreams

----------------------------------------------------------------------

INPUT: Bitstreams B_device[1 ... 20], each containing 60,000 bits

PARAMETER: Acceptance threshold alpha = 0.01

OUTPUT: Device-level p-values and pass/fail table

TESTS <- {Monobit Test, Frequency within a Block Test, Runs Test,

Longest Run of Ones in a Block Test, Binary Matrix Rank Test,

Discrete Fourier Transform Test, Non-overlapping Template Matching Test,

Serial Test, Approximate Entropy Test, Cumulative Sums Test - Forward,

Cumulative Sums Test - Backward, Random Excursions Test,

Random Excursions Variant Test}

FOR device <- 1 TO 20:

bits <- FIRST_60000_BITS(B_device[device])

FOR test IN TESTS:

p_value <- RUN_NIST_TEST(test, bits)

status <- PASS IF p_value >= alpha ELSE FAIL

STORE(device, test, p_value, status)

EXPORT complete device-level p-value and pass/fail table.

Algorithm S3. Construction of a 512-dimensional hardware-derived latent vector

------------------------------------------------------------------------------

INPUT: Transient-current traces I_device[1 ... 9], selected devices a and b, start index t0

PARAMETERS: I_min = 5 uA; I_max = 30 uA; latent bounds = [-2, 2]

OUTPUT: Latent vector z[0 ... 511]

FUNCTION NORMALIZE(I):

RETURN 4 x (I - 5 uA) / (30 uA - 5 uA) - 2

FOR k <- 0 TO 255:

z[2k]	<- NORMALIZE(I_device[a][t0 + k])

z[2k + 1] <- NORMALIZE(I_device[b][t0 + k])

RETURN z

Each vector contains 256 consecutive measured samples from each of two separately measured hardware current channels, arranged by alternating assignment.

Algorithm S4. StyleGAN2-AFHQv2 inference using the hardware-derived latent vector

---------------------------------------------------------------------------------

INPUT: Latent vector z[0 ... 511]

MODEL: NVIDIA pretrained stylegan2-afhqv2-512x512.pkl

PARAMETERS: truncation_psi = 0.7; noise_mode = constant

OUTPUT: Generated animal image

G <- LOAD_NETWORK_PICKLE(model_file)["G_ema"]

z_batch <- RESHAPE(z, shape = [1, 512])

WITH gradient computation disabled:

image <- G(z_batch, class_label = None,

truncation_psi = 0.7,

noise_mode = "const")

image <- CLAMP(image, -1, 1)

image <- (image + 1) / 2

RETURN image

The pretrained generator performs image synthesis; the mixed-ZGO measurements provide the hardware-derived latent input. The generator is used for inference without retraining or fine-tuning.

Algorithm S5. MNIST proof-of-concept workflow

---------------------------------------------

INPUT: Public MNIST training and test splits loaded through torchvision.datasets.MNIST

OUTPUT: Two-dimensional latent map and generated-digit class distribution

mnist_train <- LOAD_MNIST(train = TRUE, download = TRUE)

mnist_test <- LOAD_MNIST(train = FALSE, download = TRUE)

TRAIN a simple GAN with latent dimension 2 on the MNIST image data.

TRAIN or load a digit classifier and verify its classification accuracy.

FOR each specified noise level sigma:

SAMPLE two-dimensional latent vectors using the specified distribution.

GENERATE 1,000 digit images with the trained GAN.

CLASSIFY the generated images.

EXPORT the latent-space projection and class-frequency distribution.

This MNIST workflow is a conceptual validation of latent-space controllability and is separate from the AFHQv2 animal-image inference.

Public resources

----------------

MNIST: https://docs.pytorch.org/vision/stable/generated/torchvision.datasets.MNIST.html

AFHQv2: https://github.com/clovaai/stargan-v2

StyleGAN2-AFHQv2 model list: https://github.com/NVlabs/stylegan3

StyleGAN2-ADA PyTorch: https://github.com/NVlabs/stylegan2-ada-pytorch
